# When are Quarnets Sufficient to Reconstruct Semi-directed Phylogenetic Networks?

**DOI:** 10.1007/s11538-025-01510-5

**Published:** 2025-08-28

**Authors:** Katharina T. Huber, Leo van Iersel, Mark Jones, Vincent Moulton, Leonie Veenema - Nipius

**Affiliations:** 1 Delft Institute of Applied Mathematics, Delft University of Technology, Mekelweg 4, 2628CD Delft, The Netherlands; 2 School of Computing Sciences, University of East Anglia, NR4 7TJ Norwich, United Kingdom

**Keywords:** Quarnet, Semi-directed phylogenetic network, Level-2 network, Blob tree, Encoding

## Abstract

Phylogenetic networks are graphs that are used to represent evolutionary relationships between different taxa. They generalize phylogenetic trees since for example, unlike trees, they permit lineages to combine. Recently, there has been rising interest in *semi-directed* phylogenetic networks, which are mixed graphs in which certain lineage combination events are represented by directed edges coming together, whereas the remaining edges are left undirected. One reason to consider such networks is that it can be difficult to root a network using real data. In this paper, we consider the problem of when a semi-directed phylogenetic network is defined or *encoded* by the smaller networks that it induces on the 4-leaf subsets of its leaf set. These smaller networks are called *quarnets*. We prove that semi-directed binary level-2 phylogenetic networks are encoded by their quarnets, but that this is not the case for level-3. In addition, we prove that the so-called *blob tree* of a semi-directed binary network, a tree that gives the coarse-grained structure of the network, is always encoded by the quarnets of the network. These results are relevant for proving the statistical consistency of programs that are currently being developed for reconstructing phylogenetic networks from practical data, such as the recently developed Squirrel software tool.

## Introduction

**Fig. 1:**
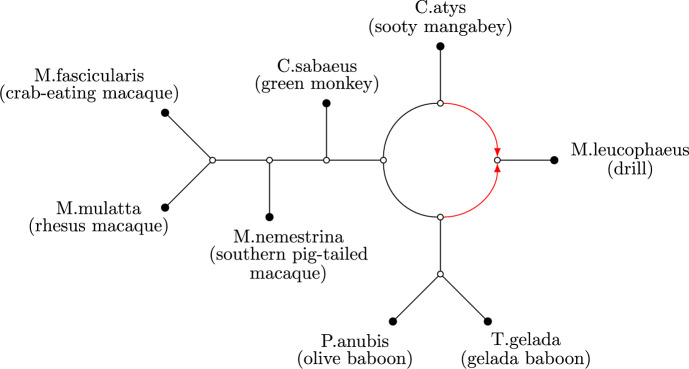
An example of a semi-directed phylogenetic network generated by the Squirrel software tool (Holtgrefe et al. 2025) for an Old World monkey dataset (Vanderpool et al. 2020) of Cercopithecinae. The edges are black and the arcs are red

*Phylogenetic networks* are graphs used to represent evolutionary relationships between different *taxa* (e.g. species, languages or other evolving objects). They are a generalization of the well-known phylogenetic trees, which are restricted to representing tree-like evolution in which lineages split but cannot combine (Bapteste et al. 2013). Both unrooted, undirected as well as rooted, directed phylogenetic networks have been and are still being studied intensively (Elworth et al. 2019; Huson et al. 2010). Recently, there has been rising interest in *semi-directed* phylogenetic networks, which are unrooted and have undirected edges as well as directed edges (for an example, see Figure 1) Allman et al. (2024); Barton et al. (2022); Huebler et al. (2019); Linz and Wicke (2023); Martin et al. (2023); Solís-Lemus et al. (2017); Wu and Solís-Lemus (2024). The reason that semi-directed networks have become more popular is that the location of the root of a network can often not be identified from real data (Kong et al. 2022). Even so, rather than reverting to completely undirected networks, semi-directed networks do permit directed edges (called *arcs*) that can be used to represent so-called *reticulations*, in which two lineages combine into one lineage that is at the end of two arcs. Such reticulations are commonly used to model reticulate evolutionary events such as hybridization, introgression, recombination or lateral gene transfer, and there are approaches that can be used to identify such events from real data (see e.g. Solís-Lemus et al. (2017)). For example, the taxon *M.leucophaeus* in Figure 1 is below two arcs which indicates a reticulation event. Essentially, semi-directed phylogenetic networks are defined as those networks that can be obtained from a directed phylogenetic network by forgetting the direction of all arcs, except for the arcs that represent reticulations, and suppressing the root.

In this paper, we study the fundamental biological question of how much information is needed to reconstruct semi-directed phylogenetic networks, a question studied for rooted, directed phylogenetic networks in Huber et al. (2015); van Iersel et al. (2022) and for unrooted phylogenetic networks in Erdős et al. (2019). More concretely, we study which semi-directed evolutionary histories can be recovered from the evolutionary histories of groups of 4 taxa (called *quarnets*). This is a topical issue since several methods have been introduced recently to generate quarnets from DNA sequences or from gene trees (Allman et al. 2025; Cummings and Hollering 2025; Holtgrefe et al. 2025; Martin et al. 2023). If a semi-directed phylogenetic network is uniquely determined by its induced subnetworks on sets of 4 taxa, then we say that the network is *encoded* by quarnets. Therefore, the question we study here can be formalized as the question of when a semi-directed phylogenetic network is encoded by its quarnets.

This question is important for at least two reasons. The first reason is algorithmic. Accurate sequence-based phylogenetic network reconstruction methods (such as maximum likelihood) are often restricted to small numbers of taxa such as quartets. Hence, in order to prove that approaches which puzzle together quarnets into a larger semi-directed phylogenetic network are correct, we need to know when quarnets encode such networks. The other reason for studying quarnet encodings is that they can be used to show identifiability results for certain classes of phylogenetic networks from sequence data that is assumed to have evolved under some evolutionary model. In particular, the main idea is to prove identifiability of quarnets using techniques from algebraic geometry, and subsequently use quarnet encodings to generalize these results to larger networks (Allman et al. 2022; Ardiyansyah 2021; Cummings et al. 2024; Gross et al. 2021).

### Previous Results

Encoding results for phylogenetic trees have been known for some time. Unrooted phylogenetic trees can be encoded by their splits, their quartets or by the distances between taxa (Dress et al. 2012). Similarly, rooted phylogenetic trees can be encoded by clusters, triplets or ultrametric distances. Distances can still be used to identify some features of certain networks (Jingcheng and Ané 2023) and some directed phylogenetic networks are still encoded by their triplets, which are 3-leaf trees contained in the network (Gambette and Huber 2012; Gambette et al. 2017). However, most networks are not encoded by their triplets. This led to research on binet, trinet and quarnet encodings (Cardona and Pons 2017; Huber and Moulton 2013; van Iersel and Moulton 2014; van Iersel et al. 2017), which are 2-leaf, 3-leaf and 4-leaf subnetworks respectively, and can be either directed, undirected or semi-directed. Note that most of the results mentioned below are restricted to binary networks (whose internal non-root vertices have total degree 3).

General directed phylogenetic networks are not encoded by their trinets (Huber et al. 2015). Hence, research has focused on encodings of subclasses of directed phylogenetic networks, e.g. by bounding their “level”. A network is *level-k* if it can be turned into a tree by deleting at most *k* edges/arcs from each blob. For example, networks $N_d$ and *N* in Figure 4 are level-2. Directed level-1 phylogenetic networks are encoded by their trinets (Huber and Moulton 2013), and so are directed level-2 phylogenetic networks and other well-studied classes: so-called directed tree-child phylogenetic networks (van Iersel and Moulton 2014) and directed orchard phylogenetic networks (Semple and Toft 2021). However, directed level-3 phylogenetic networks are not all encoded by their trinets (van Iersel et al. 2022). On the algorithmic side, it has been shown that directed level-2 and orchard phylogenetic networks can be reconstructed from all their trinets in polynomial time (Semple and Toft 2021; van Iersel et al. 2022). For directed level-1 phylogenetic networks this is also possible and, moreover, a heuristic algorithm exists that constructs directed level-1 phylogenetic networks from practical data (Oldman et al. 2016). Encoding results have been used to show that this algorithm returns the correct network if its input data consists of all trinets of a directed level-1 phylogenetic network. Unfortunately, given any set of directed trinets (not necessarily one per triple of taxa) it is NP-hard to decide whether there exists a directed phylogenetic network that contains all given trinets, already for level-1 (Huber et al. 2017).

Much less is known about encodings for semi-directed phylogenetic networks. Two algorithms for constructing a semi-directed level-1 phylogenetic network from quarnets are given in (Huebler et al. 2019) but the paper does not prove explicitly that the algorithms always reconstruct the correct network, i.e. they do not prove that semi-directed level-1 phylogenetic networks are encoded by quarnets. Nevertheless, most features of level-1 phylogenetic networks are already determined by quartets (4-leaf trees contained in the network) (Baños 2019). Moreover, recently Squirrel (Holtgrefe et al. 2025), NANUQ+ (Allman et al. 2025), Phynest (Kong et al. 2025), CUPNS (Warnow et al. 2025) and SNAQ (Solís-Lemus et al. 2017) have been introduced for generating level-1 semi-directed phylogenetic networks from quarnets, sequence alignments, SNPs and collections of gene trees.

### Our Contribution

In this paper, we study the quarnets of semi-directed phylogenetic networks. Reflecting the relative complexity of restricting a semi-directed network to a subset of its taxa, we show that this process is well-defined (see Section 4). While this is obvious for directed networks and level-1 semi-directed networks, for higher-level semi-directed networks it takes some care to prove that the intuitive definition works. Moreover, in our main result we show that semi-directed binary level-2 phylogenetic networks are encoded by their quarnets:

#### Theorem

6.2. The class of semi-directed, level-2, binary phylogenetic networks with at least 4 leaves is encoded by quarnets.

Interestingly, this is the theoretical limit for which semi-directed networks can be encoded, when categorizing networks by level. More specifically, we show that semi-directed level-3 phylogenetic networks are not all encoded by their quarnets, which shows that there are fundamental limitations for extending methods to level-3 and higher:

#### Theorem 1.1

The class of semi-directed, level-3, binary phylogenetic networks with at least 4 leaves is not encoded by quarnets.

**Fig. 2:**
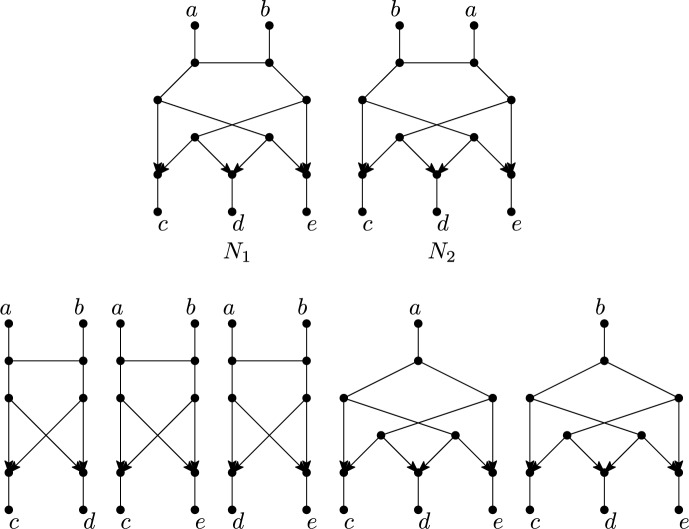
Two semi-directed level-3 phylogenetic networks $N_1$ and $N_2$ (top) and their five quarnets (bottom). Even though $N_1$ and $N_2$ have exactly the same set of quarnets, the networks themselves are not isomorphic

The above theorem can be verified easily by considering the example in Figure 2, in which an example is presented of two different networks that have the same set of quarnets. Moreover, we note that the example can be extended to any number of leaves by inserting leaves between (or next to) *a* and *b* in $N_1$ and in $N_2$ (in any order).

In order to prove our main result (Theorem 6.2) we show that the “blob tree” of a semi-directed phylogenetic network, also called the “tree of blobs”, is uniquely determined by the quarnets of the network. Basically, a “blob” of a semi-directed network is a maximal subnetwork that cannot be disconnected by deleting a single edge/arc. The blob tree of such a network is obtained by contracting each blob to a single vertex (for more details, see Section 5). Blob trees have gained interest recently, since they represent the high-level branching structure of a network and may be identifiable even when the full network is not (Allman et al. 2024, 2023; van Iersel and Moulton 2014; Rhodes et al. 2025). For all $k\ge 1$, we show that the blob tree of a semi-directed binary level-*k* phylogenetic network is always encoded by the quarnets of the network:

#### Corollary

 5.6. Suppose that *N* and $N'$ are semi-directed phylogenetic networks on *X* with the same set of quarnets. Then *N* and $N'$ have the same blob tree.

Note that this result was recently used in Holtgrefe et al. (2025) to prove that the Squirrel program correctly reconstructs level-1 networks from perfect data.

This paper is based in part on preliminary results in the MSc thesis (Nipius 2022).

### Outline of the Paper

In Section 2, we give most of the main definitions used in this paper. In Section 3, we formally define the restriction of a (semi-)directed network to a subset of leaves and show it is well-defined. Based on this, we define quarnets and quarnet encodings in Section 4, where we also show that a semi-directed level-*k* binary phylogenetic network with no non-trivial cut edges is encoded by its quarnets for $k \le 2$. In Section 5, we show that the blob tree of any semi-directed level-*k* binary phylogenetic network is encoded by its quarnets for all $k\ge 1$ or, equivalently, that the partition of the leaf set induced by a non-trivial cut edge is encoded by the quarnets. Combining the results from Sections 4 and 5, in Section 6 we show that semi-directed level-2 binary phylogenetic networks are encoded by their quarnets. In Section 7 we end with a discussion of possible future directions.

## Preliminaries

Let *X* be a finite set with $|X|\ge 2$.

We consider mixed graphs which may have undirected edges and/or directed arcs and which may have parallel arcs. Undirected edges will simply be called *edges* while directed edges will be called *arcs*. When both are possible we will write “edge/arc”. In this paper, there will be no reason to consider parallel edges or parallel edge-arc pairs. Formally, a *mixed graph* is an ordered tuple $G=(V,E)$ where *V* is a nonempty set of vertices, *E* is a multiset of undirected *edges* $\{u,v\}\subseteq V$, $u\ne v$, and directed *arcs* (*u*, *v*) with $u,v\in V$, $u\ne v$, such that each edge $\{u,v\}$ has multiplicity at most 1 in *E* and such that for all arcs $(u,v)\in E$ we have that $\{u,v\}\notin E$ and $(v,u)\notin E$. A mixed graph is *connected* if its underlying undirected graph contains a path between any two vertices. The *degree* of a vertex is the total number of incident edges and arcs. A *leaf* is a degree-1 vertex. The *indegree* of a vertex is the number of incoming arcs and the *outdegree* is the number of outgoing arcs. A *reticulation* is a vertex with indegree 2. Reticulations that are adjacent to a leaf are called *leaf reticulations*.

For a set of vertices $S \subseteq V$ in a mixed graph $G=(V,E)$ with vertex set *V* and edge/arc set *E*, an edge/arc *e* is *incident* to *S* if exactly one of its vertices is in *S*. If *e* is an arc (*u*, *v*) and $S\cap \{u,v\} = \{v\}$, we say *e* is an arc *entering* *S* or an *incoming* arc of *S*. If $S\cap \{u,v\} = \{u\}$, we say *e* is an arc *leaving* *S* or an *outgoing* arc of *S*. We also define *G*[*S*] to be the subgraph of *G* induced by *S*, i.e. the graph with vertex set *S*, an edge $\{u,v\}$ for each edge $\{u,v\}$ in *G* with $u,v\in S$ and an arc (*u*, *v*) for each arc (*u*, *v*) in *G* with $u,v\in S$.

### Directed and Semi-Directed Networks

Directed and semi-directed phylogenetic networks (defined formally below) are usually considered not to have parallel arcs or vertices of degree-2 (except for the root in directed phylogenetic networks). The restriction of a (directed or semi-directed) phylogenetic network to a subset of leaves is itself a (directed or semi-directed) phylogenetic network. However, deriving the restriction involves the repeated application of reduction rules, some of which may result in mixed graphs with parallel arcs or degree-2 vertices. For this reason, we consider a slight generalization of phylogenetic networks, simply called (directed and semi-directed) *networks* (formally defined below), and reserve the qualifier *phylogenetic* for a subclass of these graphs corresponding to the usual definition.

Since we only consider binary networks in this paper, we do not include the word binary in the names of the network types defined below. We will include the word binary in the statements of theorems to avoid confusion.

#### Definition 2.1

A *directed network* on *X* is a mixed graph $N_d$, which may have parallel arcs, with the following restrictions:

- $N_d$ has no undirected edges;
- $N_d$ has no directed cycles;
- each vertex has degree at most 3, indegree at most 2 and outdegree at most 2;
- there is a unique vertex with indegree 0, which has outdegree 2 and is called the *root*; and
- the vertices with outdegree-0 have indegree-1 and are bijectively labelled by the elements from *X*.

#### Definition 2.2

A *semi-directed network* on *X* is a mixed graph *N* that can be obtained from a directed network $N_d$ on *X* by replacing all arcs with edges except for arcs entering reticulations and subsequently suppressing the root $\rho$ if one of the following operations is applicable:

- if $\rho$ is a degree-2 vertex with incident edges $\{u,\rho \},\{\rho ,w\}$, replace these two edges by the edge $\{u,w\}$ and delete $\rho$; and
- if $\rho$ is a degree-2 vertex with an incident edge $\{u,\rho \}$ and an incident arc $(\rho ,w)$, replace this arc and edge by the arc (*u*, *w*) and delete $\rho$.

We call $N_d$ a *rooting* of *N*. If $N_d$ is a rooting of *N*, we call *N* the *underlying semi-directed network* of $N_d$ and we write $N=\overline{N_d}$.

**Fig. 3:**
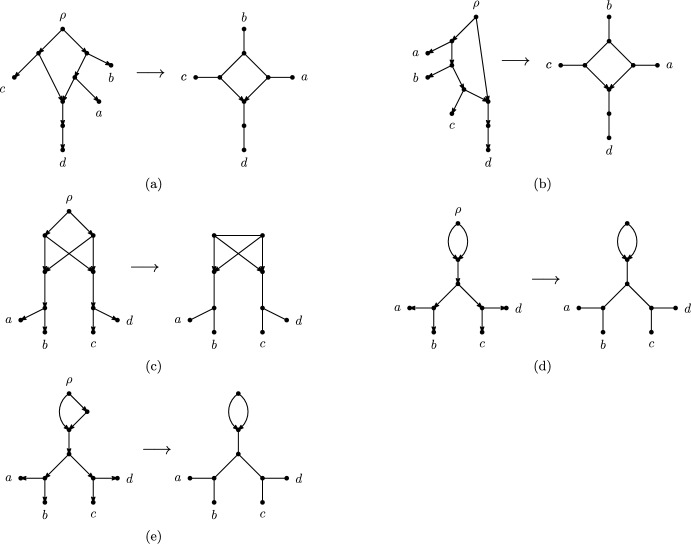
Some examples of a directed network (left) together with its underlying semi-directed network (right). Observe that the directed networks in Figure 3a and Figure 3b have the same underlying semi-directed network, as do the directed networks in Figure 3d and Figure 3e

See Figure 3 for examples of directed and semi-directed networks. We note that semi-directed networks can have more than one rooting (see for example Figures 3a and 3b). Observe that $\overline{N_d}$ is well-defined, and that if $N_{d1}$ and $N_{d2}$ are rootings of the same semi-directed network *N* then $\overline{N_{d1}}= \overline{N_{d2}} = N$. Also note that it is possible that neither of the two suppressing operations in Definition 2.2 is applicable (see Figure 3(d)).

**Fig. 4:**
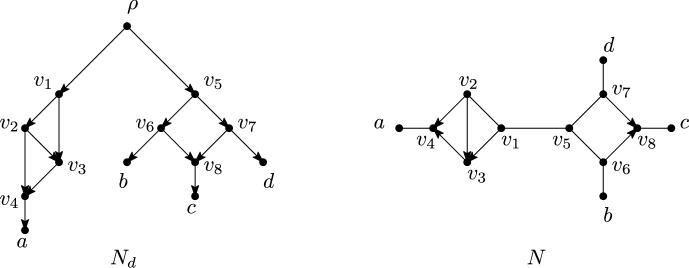
A directed network $N_d$ and its underlying semi-directed network *N*. Both $N_d$ and *N* have two blobs, each having vertex sets $\{v_1,\ldots ,v_4\}$ and $\{v_5,\ldots ,v_8\}$. Note that neither $N_d$ nor *N* is phylogenetic due to the blobs with vertex set $\{v_1,\ldots ,v_4\}$, which have 2 incident edge/arcs. Examples of $\wedge$-paths are $(a,v_4,v_3,v_1,\rho ,v_5,v_7,d)$ in $N_d$ and $(a,v_4,v_3,v_1,v_5,v_7,d)$ and $(a,v_4,v_3,v_1,v_5,v_7,v_8,c)$ in *N*. An example of a cycle, in both $N_d$ and in *N*, is $(v_1,v_2,v_4,v_3,v_1)$ with sink $v_4$

We also note that a semi-directed network *N* may have parallel arcs. This is the case if the directed network $N_d$ from which *N* is obtained has parallel arcs or has its root in a triangle (i.e., an undirected length-3 cycle), as in Figure 3d and Figure 3e.

A *network* is either a directed or a semi-directed network.

A *blob* of a mixed graph is a connected subgraph with at least three vertices that is maximal under the property that deleting any edge/arc from the subgraph does not disconnect the graph – see Figure 4 for an example. An edge/arc *e* is *incident* to a blob *B* if *e* is incident with *V*(*B*), the vertex set of *B*.

#### Definition 2.3

A network on *X* is called *phylogenetic* if

- it has no parallel arcs;
- it has no degree-2 vertices other than the root in case the network is directed; and
- it has no blobs with at most 2 incident edges/arcs, other than possibly a blob with no incoming and two outgoing arcs in case the network is directed.

See Figure 4 for an example of how a network can fail to be phylogenetic.

A (directed/semi-directed) phylogenetic network with no reticulations is called a *(rooted/unrooted) phylogenetic tree*.

We note that semi-directed phylogenetic networks as defined here do not contain any parallel arcs, even though some previous papers do allow one or more pairs of parallel arcs to be contained in such networks.

Two networks $N,N'$ on *X* are *isomorphic*, denoted $N\cong N'$, if there exists a bijection $\phi$ from the vertex set of *N* to the vertex set of $N'$ such that $\{u,v\}$ is an edge of *N* if and only if $\{\phi (u),\phi (v)\}$ is an edge of $N'$, (*u*, *v*) is an arc of *N* if and only if $(\phi (u),\phi (v))$ is an arc of $N'$ and $\phi (x)=x$ for all $x\in X$. For sets of networks $\mathcal {N},\mathcal {N'}$ on *X*, we write $\mathcal {N}\simeq \mathcal {N'}$ if there exists a bijection $\psi :\mathcal {N}\rightarrow \mathcal {N'}$ such that $N \cong \psi (N)$ for all $N\in \mathcal {N}$.

### Paths and Cycles

A *path* in a network is a sequence of pairwise distinct vertices $(v_1,\ldots ,v_p)$, $p \ge 1$, such that for all $i\in \{1,\ldots ,p-1\}$ either $(v_i,v_{i+1})$ or $(v_{i+1},v_{i})$ is an arc or $\{v_i,v_{i+1}\}$ is an edge. Such a sequence is a *semi-directed path* (from $v_1$ to $v_p$) if for all $i\in \{1,\ldots ,p-1\}$ either $(v_i,v_{i+1})$ is an arc or $\{v_i,v_{i+1}\}$ is an edge. Given two vertices *u*, *v* of a network, we say that *v* is *below* *u* if there exists a semi-directed path from *u* to *v* (possibly $u=v$). If, in addition, $u \ne v$ we say *v* is *strictly below* *u*. If *v* is (strictly) below *u* then we say *u* is *(strictly) above v*.

We now introduce $\wedge$-paths, which can be pronounced as “wedge paths”.^1^ A $\wedge$-*path* (between $v_1$ and $v_p$) in a network is a sequence of distinct vertices $(v_1,\ldots ,v_i,\ldots ,v_p)$, $p \ge 1$, such that $(v_i,\ldots ,v_1)$ and $(v_i,\ldots ,v_p)$ are semi-directed paths, for some $i\in \{1,\ldots ,p\}$ – see Figure 4 for an example. Such paths will be used when restricting a network to a subset of taxa.

A *cycle* in a network *N* is a sequence $(v_1,e_1,v_2,e_2\ldots ,v_p=v_1)$, $p \ge 4$, alternating between vertices $v_i$ and edges or arcs $e_j$ such that $v_i\ne v_j$ for $1\le i<j<p$ and for all $i\in \{1,\ldots ,p-1\}$ either $e_i=(v_i,v_{i+1})$ or $e_i=(v_{i+1},v_{i})$ is an arc of *N* or $e_i=\{v_i,v_{i+1}\}$ is an edge of *N*. We may also describe a cycle by only its vertices $(v_1,v_2,\ldots ,v_p=v_1)$. We say that a reticulation *r* in *N* is a *sink* of a cycle *C* if *C* contains both incoming arcs of *r*. See Figure 4 for an example.

A *semi-directed cycle* in a network is a cycle $(v_1,e_1,v_2,e_2\ldots ,v_p=v_1)$ such that for all $i\in \{1,\ldots ,p-1\}$ either $e_i=(v_i,v_{i+1})$ or $e_i=\{v_i,v_{i+1}\}$.

#### Lemma 2.4

In a semi-directed network *N* each cycle has at least one sink. In particular, *N* contains no semi-directed cycles.

#### Proof

Suppose *N* has a cycle $C={(v_1,v_2,\ldots ,v_p=v_1)}$ without sinks. Let $N_d$ be a rooting of *N*. Then $V(N_d) = V(N)\cup \{\rho \}$, with $\rho$ the root of $N_d$, and $N_d$ either contains a cycle $(v_1,\ldots ,v_p)$ or a cycle $(v_1,\ldots ,v_{j-1},\rho ,v_j,\ldots ,v_p)$.

First suppose that $N_d$ contains a cycle $(v_1,\ldots ,v_p)$. Since $N_d$ is acyclic, $N_d$ contains some arc $(v_{i-1},v_{i})$. Following *C* from $v_{i-1}$, at some point there is an arc $(v_{k-1},v_k)$ followed by an arc $(v_{k+1},v_k)$, again by the acyclicity of $N_d$. However, then *N* also contains arcs $(v_{k-1},v_k),(v_{k+1},v_k)$ and hence $v_k$ is a sink of *C*.

Now consider the second case, that $N_d$ contains a cycle $(v_1,\ldots ,v_{j-1},\rho ,v_j,\ldots ,v_p)$. Then we can conclude, similarly to the previous case, that $N_d$ contains arcs $(u,v_k),(w,v_k)$ with $u\in \{v_{k-1},\rho \}$ and $w\in \{v_{k+1},\rho \}$. In all cases, *N* contains arcs $(v_{k-1},v_k),(v_{k+1},v_k)$ and hence $v_k$ is a sink of *C*.

The second part of the lemma follows directly from the observation that a semi-directed cycle has no sink. $\square$

## Restricting Networks

In this section, we formally define the *restriction* $N|_A$ of a network *N* on *X* to a subset of taxa $A \subseteq X$ and consider some of its properties. In subsequent sections our focus will be on quarnets coming from a network, which are simply restrictions to subsets of size 4.

Roughly speaking, for a (phylogenetic) network *N* on *X* and a subset $A \subseteq X$, there are two main steps to constructing $N|_A$:

1. Delete all vertices that are not contained on any path between two leaves in *A*, resulting in a (not necessarily phylogenetic) network on *A*.
2. Transform this network to a phylogenetic network on *A* by repeatedly suppressing degree-2 vertices, parallel arcs, and blobs with at most 2 incident edge/arcs.

In the remainder of this section, we make the above steps precise, and show that $N|_A$ is well-defined. The main technical task is to prove the intuitively obvious but non-trivial fact that for the suppression operations described in step 2 the order does not affect the final network, which implies that the restriction is well-defined.

### Suppression Operations

**Fig. 5:**
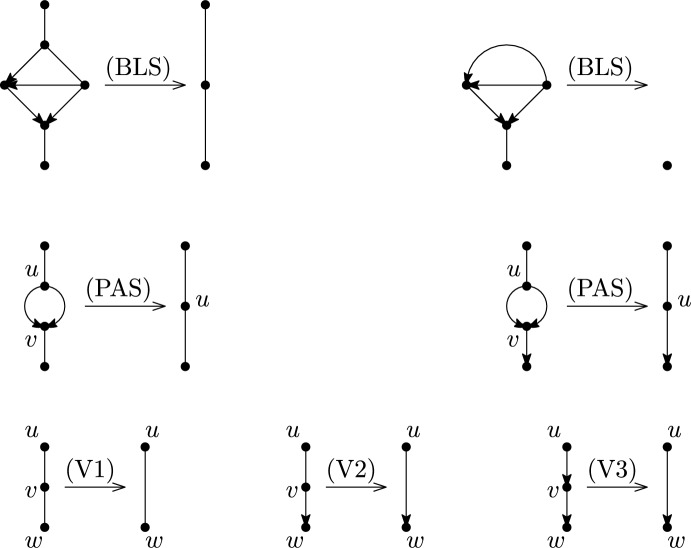
Illustrations of the suppression operations used to turn a semi-directed network into a semi-directed phylogenetic network

We now formally define the suppression operations that are used to reduce a network to a phylogenetic network. See Figure 5 for illustrations focusing on semi-directed networks.

The *blob suppression* operation on a network does the following for every blob *B* with at most two incident edges/arcs that are not two arcs leaving *B*:

(BLS): collapse *B* to a single vertex $v_B$ and, if $v_B$ has degree 1, delete it.

The *parallel arc suppression* operation on a network *N* does the following for each pair of vertices *u*, *v* with two arcs (*u*, *v*):

(PAS): if *u* and *v* both have degree 3 then remove the arcs (*u*, *v*), replace any arc (*v*, *w*) by (*u*, *w*), any edge $\{v,w\}$ with $\{u,w\}$ and delete *v*.

The *vertex suppression* operations on a network apply, for each degree-2 vertex $v\in V$, one of the following if applicable

1. if *v* has incident edges $\{u,v\},\{v,w\}$, replace them by an edge $\{u,w\}$ and delete *v*;
2. if *v* has an incident edge $\{u,v\}$ and an incident arc (*v*, *w*), replace them by an arc (*u*, *w*) and delete *v*; and
3. if *v* has incident arcs (*u*, *v*), (*v*, *w*), replace them by an arc (*u*, *w*) and delete *v*.

Note that in a directed network only operation (V3) may be applicable. Also observe that in the definition of a semi-directed network, (V1) and (V2) are applied to the root $\rho$ (and only $\rho$) after replacing arcs not entering reticulations with edges.

Note that parallel edges will never appear. To see this, recall from Lemma 2.4 that each cycle in a semi-directed network has a sink and observe that this property is preserved under each of the suppression operations. Furthermore, a degree-2 vertex *v* with an incident arc (*u*, *v*) and edge $\{v,w\}$ will never appear. To see this, observe that semi-directed networks have the property that, for each arc (*u*, *v*), *v* has indegree-2 and this property is preserved under each of the suppression operations.

It is easy to verify that if $N'$ is derived from *N* by any of (V1), (V2), (V3), (BLS), (PAS) and *N* is a directed network, then $N'$ is a directed network. The following lemma shows that this also holds for semi-directed networks, since (V3) is not applicable in semi-directed networks.

#### Lemma 3.1

Let *N* be a semi-directed network. If $N'$ is derived from *N* by a single application of (V1), (V2), (BLS) or (PAS), then $N'$ is also a semi-directed network.

The proof of Lemma 3.1 is deferred to the appendix.

The *suppression* operation on a network *N* performs first the blob suppression operation (BLS) and then repeatedly applies the parallel arc suppression operation (PAS) and the vertex suppression operations (V1),(V2),(V3) until none of them is applicable. The resulting network is denoted $\textsc {Supp}(N)$.

The proof of the following result is quite technical, and is deferred to the appendix.

#### Lemma 3.2

$\textsc {Supp}(N)$ is well-defined for any network *N*.

### Restrictions

**Fig. 6:**
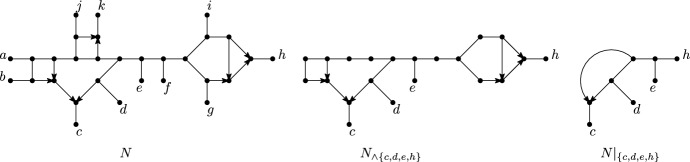
An example of restricting a semi-directed phylogenetic network *N* to a subset of the taxa $A=\{c,d,e,h\}\subseteq X$. First, all vertices are deleted that are not on a $\wedge$-path between two vertices of *A*, giving the semi-directed network $N_{\wedge A}$. Then suppression operations are applied, giving the restriction $N|_A$, which is a semi-directed phylogenetic network, by Proposition 3.4. Moreover, since $|A|=4$,  $N|_A$ is a quarnet in *Q*(*N*)

Given a network *N* on *X* and a subset $A\subseteq X$ with $|A|\ge 2$, we define $N_{\wedge A}$ as the network obtained from *N* by deleting all vertices that are not on a $\wedge$-path between two vertices in *A*. The *restriction* of *N* to *A* is defined as $N|_A=\textsc {Supp}(N_{\wedge A})$. See Figure 6 for an example. Note that for a directed network *N* it is not true in general that $\overline{N|_A}\cong \overline{N}|_A$ since suppression operations may be applicable in $\overline{N|_A}$. Consider for example the directed network *N* in Figure 3(c). Then $N|_{a,b,c,d}$ is equal to *N* and $\overline{N|_{a,b,c,d}}$ is the indicated semi-directed network. However, $\overline{N}|_{a,b,c,d}$ is an unrooted phylogenetic tree since the blob with two incident edges is suppressed.

To prove that $N|_A$ is a semi-directed phylogenetic network, if *N* is a semi-directed phylogenetic network, we will use the following lemma, whose proof is deferred to the appendix.

#### Lemma 3.3

Consider a network *N* on *X*, leaves $a,b\in X$ and a reticulation *v* with parents *u*, *w*. If *v* is on a $\wedge$-path in *N* between *a* and *b*, then *u* is on a $\wedge$-path in *N* between *a* and *b*.

#### Proposition 3.4

Given a semi-directed phylogenetic network *N* on *X* and a subset $A\subseteq X$ with $|A|\ge 2$, the restriction $N|_A$ of *N* to *A* is a semi-directed phylogenetic network.

#### Proof

We first show that $N_{\wedge A}$ is a semi-directed network. Let *D* be a rooting of *N*. Observe that a non-root vertex *v* of *D* is on a $\wedge$-path between vertices in *A* if and only if the corresponding vertex $v'$ of *N* is on a $\wedge$-path between vertices in *A*. Hence, $D_{\wedge A}$ contains all vertices of $N_{\wedge A}$ and possibly one additional vertex; its root. We split the rest of the proof into two cases accordingly.

The first case is that $D_{\wedge A}$ contains the root of *D*. In this case, $D_{\wedge A}$ contains all vertices of $N_{\wedge A}$ and exactly one additional vertex; its root $\rho$. We claim that $\overline{D_{\wedge A}}$ is equal to $N_{\wedge A}$. To prove this, it remains to show that each edge/arc has the same orientation in $\overline{D_{\wedge A}}$ as in $N_{\wedge A}$.

To this end, suppose that (*u*, *v*) is an arc of $\overline{D_{\wedge A}}$. Then *D* contains either arc (*u*, *v*) or arcs $(\rho ,u),(\rho ,v)$. In either case, since *v* is a reticulation in *D*, *N* contains an arc (*u*, *v*). Moreover, since *v* is in $N_{\wedge A}$, it follows from Lemma 3.3 that both incoming arcs of *v* in *N* are in $N_{\wedge A}$. Hence, (*u*, *v*) is an arc of $N_{\wedge A}$.

Now, suppose that (*u*, *v*) is an arc of $N_{\wedge A}$ and hence of *N*. Then *D* contains either arc (*u*, *v*) or arcs $(\rho ,u),(\rho ,v)$. In either case, *v* is a reticulation in *D*. Moreover, since *v* is in $D_{\wedge A}$, it follows from Lemma 3.3 that both incoming arcs of *v* in *D* are in $D_{\wedge A}$. Hence, $D_{\wedge A}$ contains either arc (*u*, *v*) or arcs $(\rho ,u),(\rho ,v)$. In either case, (*u*, *v*) is an arc of $\overline{D_{\wedge A}}$.

We have now shown that, in the first case, $\overline{D_{\wedge A}}$ is equal to $N_{\wedge A}$. Hence, $N_{\wedge A}$ is a semi-directed network.

Now consider the second case, i.e., that $D_{\wedge A}$ does not contain the root of *D*. In this case, $D_{\wedge A}$ contains exactly the same vertices as $N_{\wedge A}$. Hence, it follows from Lemma 3.3 that (*u*, *v*) is a reticulation arc of $D_{\wedge A}$ if and only if (*u*, *v*) is a reticulation arc of $N_{\wedge A}$. This does not imply that $D_{\wedge A}$ is a rooting of $N_{\wedge A}$ because the root may be suppressed when taking the underlying semi-directed network of $D_{\wedge A}$. Therefore, consider the directed network $D'$ obtained from $D_{\wedge A}$ by subdividing either of the arcs leaving the root. Then $\overline{D'}$ is isomorphic to $N_{\wedge A}$, proving that $N_{\wedge A}$ is a semi-directed network.

We conclude that $N_{\wedge A}$ is semi-directed in both cases. By Lemma 3.1, it now follows that $N|_A$ is semi-directed. It is also easy to see that $N|_A$ is phylogenetic, since otherwise a suppression operation would be applicable. $\square$

## Simple Level-2 Networks

We aim to understand which networks are uniquely determined by their induced set of quarnets. In this section, we shall focus on understanding this for some networks that are structurally very simple. To make this more precise, we start by presenting a formal definition of a quarnet.

A *quarnet* is a semi-directed phylogenetic network with exactly four leaves. The set *Q*(*N*) of quarnets induced by a semi-directed phylogenetic network *N* is defined as

$$ Q(N) = {\left\{ N|_A \,:\, A \subseteq X, |A|=4 \right\} }. $$

The leaf set of a quarnet *q* is denoted *L*(*q*).

Note that in case *N* is an unrooted phylogenetic tree then the quarnets of *N* are generally called *quartets*.

Let *C* be a subclass of the class of semi-directed phylogenetic networks with at least four leaves. We say that *C* is *encoded by quarnets* if for each $N\in C$ and each semi-directed phylogenetic network $N'$ on the same leaf set as *N* for which $Q(N)\simeq Q(N')$ holds, we have that $N\cong N'$. We say that *C* is *weakly encoded by quarnets* if for all $N,N'\in C$ on the same leaf sets and with $Q(N) \simeq Q(N')$ holding, we have $N\cong N'$. Clearly, if *C* is encoded by quarnets then *C* is also weakly encoded by quarnets and, as is well known, the class of unrooted phylogenetic trees is encoded by quartets (see e.g. [Dress et al. (2012), Theorem 2.7]). To help keep terminology at bay, we also say that a member of *C* is *encoded/weakly encoded by quarnets* if *C* is encoded/weakly encoded by quarnets.

**Fig. 7:**
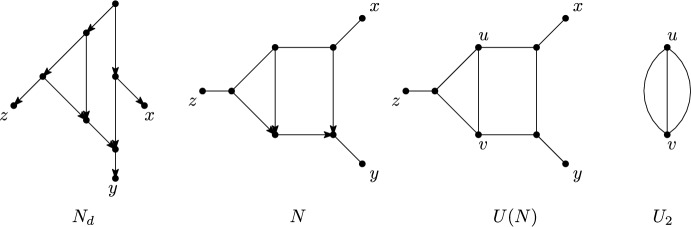
A directed, simple, strict level-2 phylogenetic network on $X=\{x,y,z\}$, a semi-directed simple, strict level-2 phylogenetic network *N* on *X*, the underlying graph *U*(*N*) of *N* and the undirected level-2 generator $U_2$

We say that a network *N* is *simple* if the mixed graph $N'$ obtained from *N* by deleting every leaf is a blob. For a non-negative integer *k* we call a network *N* *level*-*k* if each blob of *N* contains at most *k* reticulations, and we call *N* *strict level*-*k* if, in addition, it contains a blob with exactly *k* reticulations. Note that a semi-directed level-0 phylogenetic network is an unrooted phylogenetic tree in the usual sense (see e. g. Semple and Steel (2003) for more details concerning such trees) and that, by definition, a simple network is strict level-*k*, for some $k\ge 1$. For example, the directed phylogenetic network $N_d$ in Figure 7 is simple and so is the semi-directed phylogenetic network *N* in the same figure. Furthermore, both networks are strict level-2.

To be able to prove Lemma 4.1, we require further concepts. Suppose *T* is an unrooted phylogenetic tree on *X* with $|X|\ge 4$. A *cherry* in *T* is a pair of leaves of *T* that are adjacent to the same vertex of *T*. If *T* contains precisely two cherries, we call it a *caterpillar tree*.

### Lemma 4.1

The class of semi-directed, simple, strict level-1 phylogenetic networks with at least four leaves is weakly encoded by quarnets.

### Proof

Suppose that *N* is a semi-directed, simple, strict level-1 phylogenetic network with at least four leaves. Let $N'$ be a semi-directed, simple, strict level-1 phylogenetic network on the leaf set *X* of *N* with $Q(N)\simeq Q(N')$. We need to show that $N'$ is isomorphic to *N*. If $|X|=4$, this is trivial, so suppose $|X|\ge 5$.

We start with a central observation. Suppose *M* is a semi-directed, simple, strict level-1 network and *x* is a leaf of *M* that is adjacent to the unique reticulation *r* of *M*. Then, by the definition of a quarnet induced by *M*, every quarnet in *Q*(*M*) is either a semi-directed, simple, strict level-1 network such that *x* is also adjacent to *r*, or it is a phylogenetic tree whose leaf set does not contain *x*. In view of this observation, if $x \in X$ is the leaf in *N* that is adjacent to the unique reticulation in *N*, then since $Q(N)\simeq Q(N')$ it follows that *x* is adjacent to the unique reticulation in $N'$.

Now, let $P=X - \{x\}$. For every leaf $y\in P$ let $v_y$ denote the vertex in *N* adjacent with *y*. Suppose that $a,b,c,d \in P$ are such that when traversing the cycle in *N* we have the path $v_a,v_b,r=v_x,v_c,v_d$. Consider the set

$$ Q= \{ N|_A \,:\, A \in {P \atopwithdelims ()4} \}. $$

By the above observation, it is straight-forward to see that the caterpillar tree *C* on *P* with cherries $\{a,b\}$ and $\{c,d\}$ is encoded by *Q*. Since $Q(N) \simeq Q(N')$, it follows that $N'$ must induce a caterpillar tree on *P* that is isomorphic with *C*. By considering the two quarnets in *Q*(*N*) on the sets $\{a,b,x,c\}$ and $\{b,x,c,d\}$, it follows that the order of the leaves *a*, *b*, *x*, *c*, *d* in *N* induced by the path $v_a,v_b,r,v_c,v_d$ must be the same as in $N'$. Hence, $N'$ is isomorphic to *N*. $\square$

To be able to study weak encodings of level-2 networks, we refer to the graph obtained from a phylogenetic network *N* by removing all directions as the *underlying graph* of *N* and denote it by *U*(*N*), see Figure 7. Note that *U*(*N*) is indeed a graph (and not a multi-graph) because *N* is a phylogenetic network and so cannot contain parallel arcs. Note that so-called undirected phylogenetic networks are precisely the undirected graphs *G* for which there exists a semi-directed network *N* such that *G* and *U*(*N*) are isomorphic and the leaf sets of *G* and *N* coincide. Calling a multi-graph with two vertices and three parallel edges joining these vertices an *undirected level-2 generator* and canonically extending the notion of a simple, strict level-2 network to undirected phylogenetic networks then, by [van Iersel and Moulton (2018) ,Fig. 4], every undirected, simple, strict level-2 phylogenetic network on *X* can be obtained from an undirected level-2 generator by subdividing the edges of the generator to obtain three paths $P_1,P_2,P_3$ with end vertices *u* and *v* that intersect pairwise only at *u* and *v*, such that (i) at least two of these paths have length at least 2, and (ii) for $i=1,2,3$, every vertex $w \in V(P_i)\setminus \{u,v\}$ is adjacent to a leaf in *X*.

**Fig. 8:**
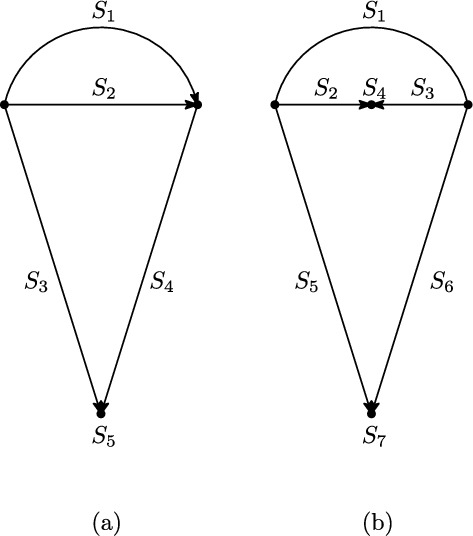
The semi-directed level-2 generators with sides labelled

Motivated by the above, we call for all $k\ge 2$ the mixed graph that can be obtained from a semi-directed, simple, strict level-*k* phylogenetic network *N* by deleting all leaves and applying vertex suppression operations (V1) and (V2) a *(semi-directed) level-k generator for N* and denote it by *gen*(*N*). More generally, we call a mixed graph *G* a *level-k generator* if there exists a semi-directed, simple, strict level-*k*, phylogenetic network *N* such that *G* and *gen*(*N*) are isomorphic. See Figure 8 for two semi-directed level-2 generators. To see that these are in fact all semi-directed level-2 generators (Lemma 4.2) we use that every semi-directed level-2 generator can be obtained from an undirected, simple, strict level-2 phylogenetic network.

### Lemma 4.2

The semi-directed level-2 generators are as pictured in Figure 8.

### Proof

Suppose that *G* is one of the mixed graphs in Figure 8. We need to show that there exists a semi-directed, simple, strict level-2, phylogenetic network *N* such that *gen*(*N*) and *G* are isomorphic. We can obtain *N* as follows. In case (a), subdivide $S_2$ into an edge, a new vertex *v*, and an arc and then add a leaf adjacent to *v*. In either case, add a leaf adjacent to each outdegree-0 reticulation. To see that *N* is semi-directed, note that you can obtain a directed network by subdividing $S_1$ by the root and directing all edges away from the root. Hence, *N* is a semi-directed, simple, strict level-2, phylogenetic network *N* such that *gen*(*N*) and *G* are isomorphic. It follows that the mixed graphs in Figure 8 are semi-directed level-2 generators.

To show that these are all semi-directed level-2 generators, consider a semi-directed, simple, strict level-2 phylogenetic network *N*. Observe that *U*(*N*) is an undirected, simple, strict level-2 phylogenetic network. Let *u* and *v* denote the vertices of the undirected level-2 generator. Let $P_1,P_2,P_3$ denote the three paths in *U*(*N*) from *u* to *v*.

Observe that *N* has, by definition, precisely two reticulations. Call these reticulations *p* and *q*. If $\{p,q\}\cap \{u,v\}=\emptyset$, then there must exist distinct $i,j\in \{1,2,3\}$ such that *p* is a vertex on $P_i$ and *q* is a vertex on $P_j$ as otherwise it would not be possible to orient the edges in *U*(*N*) so as to obtain a semi-directed, simple, strict level-2 phylogenetic network with reticulations *p* and *q*. Similarly, it is not possible that $\{p,q\}=\{u,v\}$. Hence, we must either have that $\{p,q\}=\{u,w\}$ or $\{p,q\}=\{v,w\}$, with $w \notin \{u,v\}$ a vertex on $P_i$ some $1 \le i \le 3$, or that $\{p,q\} = \{w,w'\}$ with $\{w,w'\}\cap \{u,v\}=\emptyset$ and *w* a vertex on $P_i$ and $w'$ a vertex on $P_j$, where $i,j\in \{1,2,3\}$. In the first case, *gen*(*N*) is the mixed graph in Figure 8(a). In the second case, *gen*(*N*) is the mixed graph in Figure 8(b). $\square$

As we shall see, the next result (Proposition 4.3) is central for showing that the class of semi-directed simple, strict level-2 phylogenetic networks with at least four leaves is weakly encoded by quarnets (Theorem 4.5). To be able to state and prove it, we again require further definitions.

**Fig. 9:**
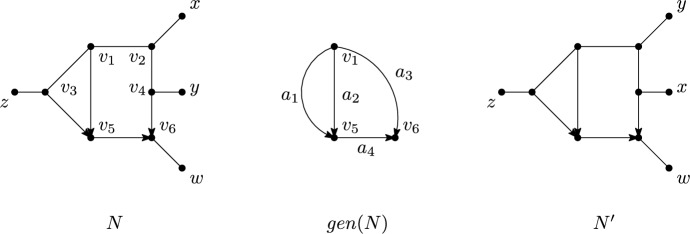
Two semi-directed, simple, strict level-2 phylogenetic networks *N* and $N'$ that are isomorphic up to sides, together with their level-2 generator $gen(N)\cong gen(N')$

The following definitions are illustrated in Figure 9. Suppose that *N* is a semi-directed, simple, strict level-2 phylogenetic network. Then we call the arcs, edges and the degree-2 vertices in *gen*(*N*) (which have indegree 2 outdegree 0) the *sides* of *gen*(*N*). For example, the sides of *gen*(*N*) in the example are the arcs $a_1,a_2,a_3,a_4$ and the vertex $v_6$. If a side *S* of *gen*(*N*) is an arc/edge, then we denote by *P*(*S*) the semi-directed path in *N* such that when deleting all leaves of *N* adjacent with a vertex of *P*(*S*) and suppressing all resulting vertices of *P*(*S*) with overall degree two, we obtain *S*. In the example, we have $P(a_3)=(v_1,v_2,v_4,v_6)$, $P(a_1)=(v_1,v_3,v_5)$ and $P(a_2)=(v_1,v_5)$ (where $a_1,a_2$ could be swapped). Note that *P*(*S*) could be an arc/edge in *N* (such as $P(a_4)=(v_5,v_6)$ . In case *S* is a vertex, then we also refer to *S* as *P*(*S*) (e.g. $P(v_6)=v_6$ . We say that a leaf *x* of *N* is *hanging off S in N* if either *S* is a vertex of *gen*(*N*) with overall degree two and *N* contains the edge $\{S,x\}$ or *S* is an arc/edge in *gen*(*N*) and there exists a vertex *v* on *P*(*S*) such that $\{v,x\}$ is an edge of *N*. In the example, *z* is hanging off $a_1$ and *w* is hanging off $v_6$. We denote the set of leaves of *N* hanging off *S* by $P_S$. In the example, $P_{a_3}=\{x,y\}$ and $P_{a_4}=\emptyset$. Finally, we say that two semi-directed, simple, strict level-2 phylogenetic networks *N*, $N'$ are *isomorphic up to sides* if there is some (mixed graph) isomorphism $\phi$ between *gen*(*N*) and $gen(N')$ so that for any side *S* in *gen*(*N*), the leaf sets $P_S$ and $P_{\phi (S)}$ are equal. In the example, *N* and $N'$ are isomorphic up to sides.

### Proposition 4.3

Suppose that *N* and $N'$ are semi-directed, simple, strict level-2 phylogenetic networks with at least four leaves that are isomorphic up to sides. If $Q(N)\simeq Q(N')$, then $N\cong N'$.

### Proof

Suppose that $Q(N)\simeq Q(N')$ and that $\phi$ is an isomorphism from *gen*(*N*) to $gen(N')$. By Lemma 4.2 it follows that *gen*(*N*) and $gen(N')$ are either both as depicted in Figure 8(a) or they are both as depicted in Figure 8(b).

**Claim:** Suppose *S* is a side of *gen*(*N*) for which $P_S\not =\emptyset$. If *S* is an arc then, irrespective of Case (a) or (b) holding for *N* in Figure 8, the order in which the elements in $P_S$ hang off $\phi (S)$ in $N'$ relative to the direction of $\phi (S)$ is the same as the order in which they hang off *S* in *N* relative to the direction of *S*. If *S* is the unique edge in Figure 8(b), then the order in which the elements in $P_S$ hang off $\phi (S)$ in $N'$ is the same as the order in which they hang off *S* in *N*, up to reversing the whole ordering.

*Proof of Claim:* Suppose *S* is an arc in *gen*(*N*) such that $P:=P_S\not =\emptyset$. If $|P|=1$, then the claim trivially holds. So assume that $|P|\ge 2$. We distinguish between the cases that $|P|=2$, that $|P|=3$, and that $|P|\ge 4$.

If $|P|=2$, then we consider a 4-subset *A* containing *P* which is defined as follows. If Figure 8(a) holds, then *N* has a unique reticulation. Let $x \in X$ be the leaf below that reticulation and let $y \in X-({P} \cup \{x\})$. If Figure 8(b) then *N* has two reticulations. Let $x,y \in X$ be the leaves below the two reticulations, respectively. In either case, let $A=P \cup \{x,y\}$. Then $N|_A\in Q(N)\simeq Q(N')$. That the claim holds is straight-forward to see.

If $|P|=3$, then we consider two 4-subsets *A*, *B* of *X* which are defined as follows. Suppose first that *gen*(*N*) is as in Figure 8(a) and that *x* is the leaf of *N* below the unique reticulation *r* of *N*. Then the size of $A:=P\cup \{x\}$ is four since $|P|=3$. Moreover, if *P* equals $P_{S_3}$ or $P_{S_4}$, then we choose a leaf *a* in $P_{S_1}$ or $P_{S_2}$ which must exist as *N* is strict level-2. To obtain *B*, we choose leaves *b*, *c* in *P* such that the unique vertex in *N* adjacent with *b* is adjacent with *r* as well as with the unique vertex in *N* adjacent with *c*. Finally, we put $B=\{a,b,c,x\}$.

Assume for the remainder of this case that *gen*(*N*) is as in Figure 8(b). Let *x*, *y* be the leaves of *N* such that *x* is below one reticulation of *N* and *y* is below the other. Then we put $A=P \cup \{x\}$ and $B= P \cup \{y\}$ which both clearly have size four since $|P|=3$.

In either of the above two cases, $N|_A, N|_B \in Q(N)\simeq Q(N')$ follows. That the claim holds is a straight-forward consequence.

If $|P|\ge 4$, then consider the set *R* of *quartets* obtained by restricting *N* to all possible 4-subsets of *P*. Then *R* must be the set of quartets induced by some caterpillar tree *T* with leaf set *P*. Since $Q(N)\simeq Q(N')$ it follows that the leaves in *P* are hanging off *S* in *N* in the same ordering as the leaves of *P* are hanging off $\phi (S)$ in $N'$, up to reversal of the two leaves in each of the cherries in *T* and up to reversing the whole ordering. The claim now follows by considering, in addition to *R*, the set of all quarnets with leaf set $\{a,b,c,x\}$, where *a* and *b* form a cherry in *T*, $c \in P- \{a,b\}$ and *x* is a leaf below a reticulation in *N*. This completes the proof of the Claim.

If *gen*(*N*) is as in Figure 8(a), then the lemma follows by applying the Claim to each side *S* of *gen*(*N*) for which $P_S\not =\emptyset$ holds. If *gen*(*N*) is as in Figure 8(b), then the lemma follows again by applying the Claim to each side *S* of *gen*(*N*) for which $P_S\not =\emptyset$ holds in case $P_{S_1}=\emptyset$, that is, no leaf of *N* is hanging off $S_1$ in *N*. Furthermore, the lemma follows by applying the Claim to side $S_1$ of *gen*(*N*) if $P_{S_i}=\emptyset$ holds for all $i\in \{2,3,5, 6\}$, that is, other than the leaves of *N* hanging off the two reticulations of *N*, every leaf of *N* is hanging off $S_1$ in *N*.

Assume for the remainder that $P_{S_1}\not =\emptyset$ and that there exists some $i\in \{2,3,5,6\}$ such that $P_{S_i}\not =\emptyset$. To see that the order in which the elements in $P_{S_1}$ are hanging off $\phi (S_1)$ in $N'$ is the same as the order in which they are hanging off $S_1$ in *N*, we may assume without loss of generality that $i=2$. Choose leaves $a\in P_{S_1}$ and $b\in P_{S_2}$ such that there exists a vertex *w* in *N* such that the shortest path from *a* to *b* in *N* contains *w*. Since *N* is a semi-directed, simple, strict level-2 network there must exist a leaf *x* of *N* that is adjacent with one reticulation of *N* and a leaf *y* of *N* that is adjacent with the other. Then $N|_{\{a,b,x,y\}}$ is a quarnet in $Q(N)\simeq Q(N')$. Thus, the shortest path from *a* to *b* in $N'$ contains $\phi (w)$. Since, by the Claim, the order in which the elements in $P_{S_1}$ are hanging off $\phi (S_1)$ in $N'$ is the same as the order in which they are hanging off $S_1$ in *N*, up to reversing the whole ordering, it follows that $N\cong N'$. This completes the proof of the lemma. $\square$

### Lemma 4.4

The class of semi-directed, simple, strict level-2 phylogenetic networks with at least four leaves is weakly encoded by quarnets.

### Proof

Suppose that *N* is a semi-directed, simple, strict level-2 phylogenetic network with at least four leaves. Let *X* be the leaf set of *N* and let $N'$ be a semi-directed, simple, strict level-2 phylogenetic network on *X* such that $Q(N) \simeq Q(N')$. We need to show that *N* and $N'$ are isomorphic. By Lemma 4.3, it suffices to show that *N* and $N'$ are isomorphic up to sides.

First note that *N* and $N'$ must have isomorphic generators. Indeed, there must be some 4-subset *A* of *X* so that $N|_A$ (and thus $N'|_A$) is a semi-directed, simple, strict level-2 phylogenetic network. Since $Q(N)\simeq Q(N')$ it follows that $gen(N|_A)$ and $gen(N'|_A)$ are isomorphic. By Lemma 4.2, it follows that *gen*(*N*) and $gen(N')$ must be isomorphic, as required.

We next show that there exists some isomorphism $\phi$ from *gen*(*N*) to $gen(N')$ so that if *S* is any side in *gen*(*N*) with $P_S\not =\emptyset$ then $P_S=P_{\phi (S)}$.

To show that such an isomorphism $\phi$ exists, we distinguish between the cases that *gen*(*N*) is as in Figure 8(a) and that *gen*(*N*) is as in Figure 8(b). Put $P_i=P_{S_i}$, for all *i*.

Case (a): Note that in this case, there are exactly two isomorphisms from *gen*(*N*) to $gen(N')$: the identity and one that swaps the sides $S_1$ and $S_2$ of *gen*(*N*). Now, fix $x\in P_5$ and an arbitrary leaf $y\in P_i$ with $i\in \{1,2\}$. Then considering any quarnet containing leaves *x* and *y* we see that *y* hangs off $\phi (S_1)$ or off $\phi (S_2)$ in $N'$, for any isomorphism $\phi$. Choose $\phi$ such that *y* hangs off $\phi (S_i)$. Now consider any leaf $z\in P_i\setminus \{y\}$, $i\in \{1,2,3,4\}$. Then considering any quarnet whose leaf set contains leaves *x*, *y* and *z*, we see that *z* hangs off $\phi (S_i)$ in $N'$. This completes the proof in this case.

Case (b): Note that in this case there are exactly four isomorphisms from *gen*(*N*) to $gen(N)'$: the identity, $(S_2S_3)(S_5S_6)$, $(S_2S_5)(S_4S_7)(S_3S_6)$ and $(S_2S_6)(S_4S_7)(S_3S_5)$ (given as a combination of swaps, where $(S_i,S_j)$ denotes swapping sides $S_i$ and $S_j$). Now, let $x\in P_4$ and $y\in P_7$. First suppose $P_1=X\setminus \{x,y\}$. For any leaf $z\in P_1$ it follows, by considering an arbitrary quarnet whose leaf set contains *x*, *y* and *z* that *z* hangs off $\phi (S_1)=S_1$ in $N'$. So the lemma holds in this case.

Assume for the remainder that there exists $q\in X\setminus (P_1\cup \{x,y\})$. Then $q\in P_i$ with $i\in \{2,3,5,6\}$. Hence, by considering any quarnet whose leaf set contains *x*, *y* and *q*, we see that *q* hangs off one of $\phi (S_2)$, $\phi (S_3)$, $\phi (S_5)$, $\phi (S_6)$ in $N'$. Choose the isomorphism $\phi$ such that *q* hangs off $\phi (S_i)$ in $N'$. Then, for any leaf $z\in P_i$, $i\in \{1,2,3,5,6\}$, it follows, by considering the quarnet whose leaf set contains *x*, *y*, *z*, *q*, that *z* hangs off $\phi (S_i)$ in $N'$. This completes the proof of the lemma in this case too. $\square$

The next theorem corresponds to Lemma 4.4 without the “strict” restriction.

### Theorem 4.5

The class of semi-directed, simple, level-2, binary phylogenetic networks with at least four leaves is weakly encoded by quarnets.

### Proof

Suppose that *N* and $N'$ are semi-directed, simple, level-2 phylogenetic networks on *X* with $Q(N)\simeq Q(N')$. We want to show that *N* is isomorphic to *N*.

First note that, if *N* is strict level-2 and $N'$ is strict level-1, then we can clearly pick some $A\subseteq X,|A|=4$, so that the quarnet $N|_A$ is a strict level-2 network, which is impossible since $N'|_A$ must be a level-1 network. By symmetry, it follows that both *N* and $N'$ must be a strict level-1 or a strict level-2 network. The theorem now follows immediately by applying Lemmas 4.1 and 4.4. $\square$

## Blob Trees

**Fig. 10:**
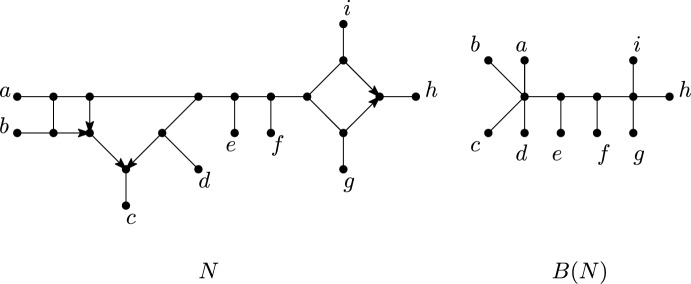
A semi-directed phylogenetic network *N* and the blob tree *B*(*N*) of *N*

Observe that a directed (respectively semi-directed) network is phylogenetic precisely if it has no parallel arcs and contracting each blob into a single vertex gives a directed (respectively undirected) phylogenetic tree. The tree obtained in this way is called the *blob tree* *B*(*N*) of a network *N*, see Figure 10. In this section, we show that the blob tree of a semi-directed phylogenetic network is uniquely determined by the quarnets of the network. This will be a direct consequence of Theorem 5.1, which characterizes the splits of a semi-directed phylogenetic network using its quarnets. Note that this theorem does not put any restriction on the level.

A *cut-edge* of a semi-directed network is an edge whose removal disconnects the network. We call a bipartition $\{A,B\}$ of *X* into two non-empty subsets *A* and *B* a *split* of *X* and denote it by *A*|*B* where the order of *A* and *B* does not matter. We call a split *A*|*B* trivial if $|A|=1$ or $|B|=1$.

Given a semi-directed network *N* on *X* and a split *A*|*B* of *X* we say that *A*|*B* is a *cut-edge split (CE-split)* in *N* if there exists a cut-edge $\{u,v\}$ of *N* such that its removal gives two connected mixed graphs with leaf-sets *A* and *B*. We say a CE-split *A*|*B* is *trivial* if $|A|=1$ or $|B|=1$. Observe that a semi-directed phylogenetic network is simple if and only if it has no nontrivial CE-splits.

We will show in this section that we can detect splits in a semi-directed phylogenetic network by looking at its quarnets, using the following theorem:

### Theorem 5.1

Let *N* be a semi-directed, binary

phylogenetic network on *X* and *A*|*B* a split of *X*. Then *A*|*B* is a CE-split in *N* if and only if one of the following holds:

- *A*|*B* is a trivial split of *X*; or
- *A*|*B* is non-trivial and for any pairwise distinct elements $a_1,a_2 \in A, b_1,b_2 \in B$, $\{a_1,a_2\}|\{b_1,b_2\}$ is a CE-split in $N|_{\{a_1,a_2,b_1,b_2\}}$.

The main challenge in proving Theorem 5.1 will be to show that when *A*|*B* is non-trivial and *N* is simple (and therefore *A*|*B* is not a CE-split in *N*), there exist $a_1,a_2 \in A, b_1,b_2 \in B$ for which $\{a_1,a_2\}|\{b_1,b_2\}$ is *not* a CE-split in $N|_{\{a_1,a_2,b_1,b_2\}}$. To show this, we first prove some results concerning directed networks:

### Lemma 5.2

Let *N* be a simple directed phylogenetic network on *X* with at least one reticulation. If *v* is a vertex of *N* that is not the root, not a leaf and not a leaf-reticulation, then

there exists an arc $(u',v')$ with $v \notin \{u',v'\}$ such that $v'$ is below *v* and $u'$ is not below *v*. In particular, $v'$ is a reticulation.

### Proof

Let *Y* denote the set of non-leaf vertices in *N* that are not below *v*, and let *Z* denote the set of non-leaf vertices in *N* strictly below *v*. Since *v* is not the root, *Y* is nonempty. In addition, since *v* is not a leaf and not a leaf-reticulation, *Z* is nonempty. Then since *N* is simple, the underlying undirected graph of *N* has a path starting at a vertex *Y* and ending at a vertex in *Z* that does not include *v*. It follows that there exist adjacent vertices $u'$ in *Y*, $v'\in Z$. Since $v'$ is below *v* and $u'$ is not, *N* does not contain the arc $(v',u')$. So *N* must contain the arc $(u',v')$, as required. $\square$

### Lemma 5.3

Let *N* be a simple directed strict level-*k* phylogenetic network on *X* for $k \ge 1$. Then for any arc (*u*, *v*) in *N* with *v* not a leaf, there exist vertices $u^*, r$ and directed paths *P*, *Q* in *N* such that:

- *P* and *Q* are arc-disjoint paths from $u^*$ to *r*;
- *P* contains the arc (*u*, *v*); and
- *r* is a leaf-reticulation.

### Proof

Suppose first that *v* is a leaf-reticulation, and let $(u',v)$ be the other incoming arc of *v*. Then let $r = v$ and let $u^*$ be any lowest common ancestor of *u* and $u'$. Let *P* be a directed path consisting of a directed path from $u^*$ to *u* extended with the arc (*u*, *v*), and let *Q* be a directed path consisting of a directed path from $u^*$ to $u'$ extended with the arc $(u',{v})$. Then *P* and *Q* are arc-disjoint paths from $u^*$ to $r=v$ (any overlap would imply that *u* and $u'$ have a common ancestor strictly below $u^*$) and *P* contains (*u*, *v*).

Now assume that *v* is either a tree node or reticulation that is not a leaf-reticulation. We generate a sequence of vertices $v_1,u_1,\dots ,v_{s-1},u_{s-1}, v_s$, as follows. Initially set $v_1: =v$ and $i = 1$. While $v_i$ is not a leaf-reticulation, by Lemma 5.2 there exists at least one arc $(u',v')$ with reticulation $v'$ strictly below $v_i$ and $u'$ not below $v_i$. Choose such an arc $(u',v')$ with lowest $v'$, and let $u_i:= u'$, $v_{i+1} = v'$. Observe that any directed path from $v_i$ to $v_{i+1}$ is arc-disjoint from any directed path ending with $(u_i,v_{i+1})$. Now increase *i* by 1 and repeat. If $v_i$ is a leaf-reticulation, then set $s: = i$ and terminate.

**Fig. 11:**
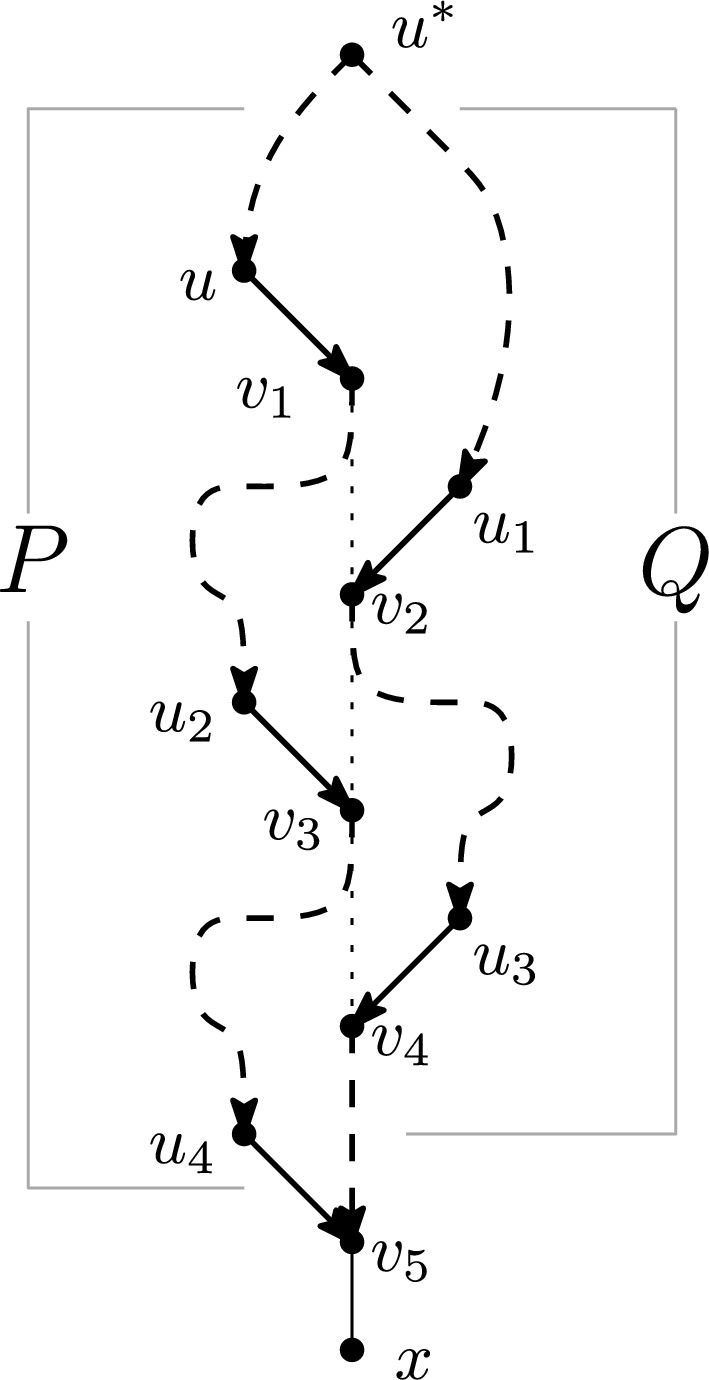
Illustration of the proof of Lemma 5.3 for the case that $s = 5$. For each $i < s$, $v_{i+1}$ is a lowest vertex below $v_i$ such that $v_{i+1}$ has a parent $u_i$ that is not below $v_i$. Dashed lines represent directed paths. The path *P* is in bold on the left, starting at $u^*$, passing through $u,v_1,u_2,v_3,u_4$ and ending at $v_5$. The path *Q* is in bold on the right, starting at $u^*$, passing through $u_1,v_2,u_3,v_4$ and ending at $v_5$. A dotted line from $v_i$ to $v_{i+1}$ illustrates the fact that $v_{i+1}$ is below $v_i$, for $i \in \{1,\dots , s-2\}$

Since $v_{i+1}$ is strictly below $v_i$ for each *i*, this process must terminate because *N* only has finitely many vertices. Let *x* be the leaf adjacent to $v_s$ — see Figure 11.

Note that $v_j$ is below $v_i$ for all $1\le i < j \le s$. Note also that for each $i \in \{1,\dots , s-2\}$, the vertex $u_{i+1}$ is below $v_i$. Indeed, if this is not the case then $(u_{i+1}, v_{i+2})$ is an arc with $v_{i+2}$ below $v_i$ and $u_{i+1}$ not below $v_i$, which contradicts our choice of $v_{i+1}$ as a lowest vertex for which such an arc exists. So there exists a path from $v_i$ to $u_{i+1}$ for each $i \le s-2$, and an arc from $u_i$ to $v_{i+1}$ for each $i \le s-1$.

Now let $u^*$ be a lowest common ancestor of *u* and $u_1$.

We can now form *P* by combining the following directed paths — see Figure 11:

- A directed path from $u^*$ to *u*;
- The arc $$(u,v_1)$$;
- For each odd $i \in \{1, \dots , s-2\}$, a directed path from $v_i$ to $u_{i+1}$;
- For each even $i \in \{1,\dots , s-1\}$, the arc $(u_i,v_{i+1})$;
- If *s* is even, a directed path from $v_{s-1}$ to $v_s$.

We now have that *P* contains $v_i$ for all odd *i* and $u_i$ for all even *i*, and *P* is a directed path from $u^*$ to $v_s$ (ending with the arc $(u_{s-1},v_s)$ if *s* is odd, and otherwise ending with an arbitrary path from $v_{s-1}$ to $v_s$). By construction, *P* contains the arc (*u*, *v*).

In a similar way, we form *Q* by combining the following directed paths — see Figure 11:

- An (arbitrary) directed path from $u^*$ to $u_1$;
- For each even $i \in \{1, \dots , s-2\}$, an (arbitrary) directed path from $v_i$ to $u_{i+1}$;
- For each odd $i \in \{1,\dots , s-1\}$, the arc $(u_i,v_{i+1})$;
- If *s* is odd, an (arbitrary) directed path from $v_{s-1}$ to $v_s$.

We now have that *Q* contains $v_i$ for all even *i* and $u_i$ for all odd *i*, and *Q* is a directed path from $u^*$ to $v_s$ (ending with the arc $(u_{s-1},v_s)$ if *s* is even, and otherwise ending with an arbitrary directed path from $v_{s-1}$ to $v_s$).

Letting *r* be the leaf-reticulation $v_s$, we have that *P* and *Q* are paths from $u^*$ to *r*. It remains to show that *P* and *Q* are arc-disjoint. For this, it is sufficient to show that there is no vertex $v'$ in both *P* and *Q* except for the $u^*$ and $v_s$. We note that the degenerate case that *P* and *Q* both consist of the single arc $(u^*,v_s)$ cannot occur, since we assumed $v_1$ is not a leaf-reticulation and so $s>1$.

So suppose for a contradiction that such a vertex $v'$ does exist. Then $v'$ is strictly below $u^*$ and strictly above $v_s$.

First suppose that $v'$ is strictly above $v_1$, and therefore $v_2$. Since $v'$ is on *P*, this implies that $v'$ is also above *u*.

Since $v'$ is on *Q*, it follows that $v'$ is above $u_1$. Thus $v'$ is a common ancestor of *u* and $u_1$ that is strictly below $u^*$, a contradiction by the choice of $u^*$.

Now suppose that $v'$ is below $v_1$. Let $i \in \{1,\dots , v_{s-1}\}$ be the unique index such that $v'$ is below $v_i$ but not below $v_{i+1}$. Since one of the paths *P* and *Q* contains $(u_i, v_{i+1})$, $v'$ must be above $u_i$. But then we have that there is a directed path from $v_i$ to $u_i$ via $v'$. Thus $u_i$ is below $v_i$, a contradiction by the choice of $u_i$. Thus we may conclude that *P* and *Q* have no vertices in common except for $u^*$ and $v_s$ (and do not both consist of a single arc), and so *P* and *Q* are arc-disjoint. $\square$

**Fig. 12:**
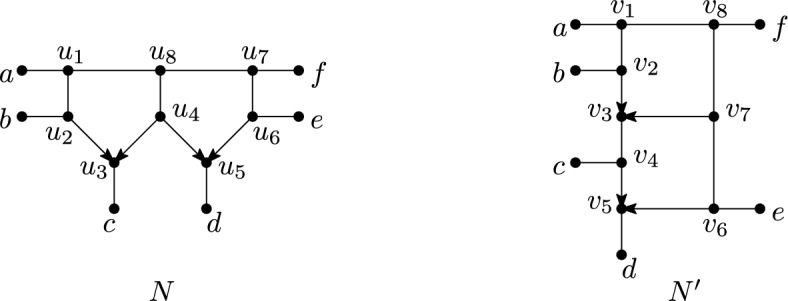
Two semi-directed phylogenetic networks *N* and $N'$, each containing three pairwise-overlapping cycles. In *N*, the cycle $(u_1, u_2, u_3, u_4, u_8, u_1)$ is good as it has a single sink $u_3$, and it is excellent as $u_3$ is adjacent to a leaf. Similarly, the cycle $(u_4, u_5, u_6, u_7, u_8, u_4)$ is excellent. However, the cycle $(u_1, u_2, u_3, u_4, u_5, u_6, u_7, u_8,u_1)$ is not good (and therefore not excellent) as it has two sinks $u_3$ and $u_5$. In $N'$, the cycle $(v_1,v_2,v_3,v_7,v_8, v_1)$ is good as it has a single sink $v_3$, but it is not excellent since $v_3$ is not adjacent to a leaf. The cycles $(v_3,v_4,v_5,v_6,v_7,v_3)$, and $(v_1,v_2,v_3,v_4,v_5,v_6,v_7,v_8,v_1)$ are both excellent. Note in particular that this last cycle is good even though it contains two reticulations $v_3$ and $v_5$, as $v_3$ is not a sink of this cycle

We say that two cycles in *N* *overlap* if they have at least one vertex in common. Since *N* is binary, two cycles in *N* overlap if and only if they have at least one edge or arc in common. Recall that a reticulation *r* in *N* is a *sink* of a cycle *C* if *C* contains both incoming arcs of *r*. We call a cycle *C* *good* if it contains exactly one sink, and we call a good cycle *excellent* if its sink is adjacent to a leaf. See Figure 12.

We say a leaf *belongs* to a cycle *C* if the unique vertex that is adjacent to it is in *C*. Note that if *r* is the sink of a good cycle *C* in a semi-directed network *N* and *x* is a leaf below *r*, then *x* belongs to *C* if and only if *r* and *x* are adjacent. To see this, suppose that *x* is a leaf below *r* and belongs to *C* but is not adjacent to *r*. Then there exists a semi-directed path from *r* to the vertex *v* adjacent to *x*. Since *v* is a vertex of *C* and *r* is the unique sink of *C*, there exists a semi-directed path from *v* to *r*. Hence, there exists a semi-directed cycle in *N*, which is a contradiction by Lemma 2.4.

### Lemma 5.4

Let *N* be a simple, semi-directed phylogenetic network with at least one reticulation and let *e* be an arc or edge between two non-leaf vertices. Then *e* is contained in at least one excellent cycle.

### Proof

Let $v_1,v_2$ be the vertices of *e*. Let $N_d$ be a rooting of *N* with root $\rho$.

Observe that either $v_1$ and $v_2$ are adjacent in $N_d$, or $N_d$ contains the arcs $(\rho , v_1), (\rho , v_2)$ (and $\rho$ is not adjacent to any other vertices). If $v_1$ and $v_2$ are adjacent in $N_d$, we may assume without loss of generality that the arc between them is $(v_1,v_2)$.

Now, let $(u,v) = (v_1,v_2)$ if $v_1$ and $v_2$ are adjacent in $N_d$, and let $(u,v) = (\rho , v_1)$ otherwise. By Lemma 5.3 there exist arc-disjoint directed paths *P*, *Q* in $N_d$ from some vertex $u^*$ to a leaf-reticulation *r*, and (*u*, *v*) is on the path *P*. Note that either $u^* = \rho$ or every vertex in *P* and *Q* is a vertex of *N*.

We now construct a cycle *C* in *N* from the union of *P* and *Q*. For each arc $e'$ in *P* or *Q* not incident to $\rho$, let $e''$ be the corresponding edge or arc in *N* (i.e. with the same vertices as $e'$), and add $e''$ to *C*. If $u^* = \rho$, then $(\rho ,v_1)$ and $(\rho , v_2)$ are the first arcs of *P* and *Q* and add the arc or edge in *N* between $v_1$ and $v_2$ to *C*. Since *P* and *Q* are arc-disjoint paths with the same start and end vertices, the resulting *C* is indeed a cycle. Moreover *C* contains *e* (either because $(v_1,v_2)$ is an arc in *P*, or because $(\rho , v_1)$ and $(\rho , v_2)$ are the top arcs of *P* and *Q* respectively). It remains to show that *C* is an excellent cycle.

To see that *C* is a good cycle, observe that any sink in *C* must have two incoming arcs in the union of *P* and *Q*. But as *P* and *Q* are edge-disjoint directed paths in $N_d$ there is only one vertex for which this holds, namely *r*. Thus *C* has only one sink. Finally, as *r* is a leaf-reticulation, there is a leaf adjacent to *r* and so *C* is excellent. $\square$

### Lemma 5.5

Let *N* be a simple, semi-directed phylogenetic network on *X* with at least one reticulation, and let *A*|*B* be any bipartition of *X*. Then there exist excellent cycles $C_1, C_2$ (possibly with $C_1=C_2$) and leaves $a \in A, b \in B$ such that $C_1$ and $C_2$ overlap and *a* belongs to $C_1$ and *b* belongs to $C_2$. In addition, either $C_1\ne C_2$ and *a* and *b* are both adjacent to a reticulation or $C_1= C_2$ and one of *a* and *b* is adjacent to a reticulation.

### Proof

Take any $a' \in A$ and $b' \in B$. Let $v_a$ be the non-leaf vertex adjacent to $a'$ and $v_b$ the non-leaf vertex adjacent to $b'$. Since *N* is connected, there exists a path (not necessarily semi-directed) between $v_a$ and $v_b$, and all vertices on this path are non-leaf vertices. Let $v_1 = v_a,v_2,\dots , v_s = v_b$ be the vertices of this path, and let $e_i$ be the edge or arc between $v_i$ and $v_{i+1}$, for each $i \in \{1,\dots , s-1\}$. By Lemma 5.4, for each $i \in \{1,\dots , s-1\}$ there exists an excellent cycle $C_i'$ containing $e_i$. As each $C_i'$ is an excellent cycle, it has at least one leaf in *A* or *B* belonging to it (namely the leaf adjacent to its sink). Note that in particular $a'$ belongs to $C_1'$ since $C_1'$ contains $v_a$, and $b'$ belongs to $C_{s-1}'$ since $C_{s-1}'$ contains $v_b$. Therefore there exists some $i \in \{1,\dots , s-2\}$ such that a leaf *a* in *A* belongs to $C_i'$, and a leaf *b* in *B* belongs to $C_{i+1}'$. Furthermore $C_i'$ and $C_{i+1}'$ must overlap, as they both contain the vertex $v_{i+1}$. Then $C_i'$ and $C_{i+1}'$ are the desired excellent cycles.

Finally, note that we can choose *a* and *b* to be both adjacent to a reticulation unless the leaves adjacent to the sinks of the $C_i'$ are all in *A* or all in *B*. If they are all in *A*, then we can take $C_1=C_2=C_{s-1}'$ and *a* is adjacent to a reticulation. If the leaves adjacent to the sinks of the $C_i'$ are all in *B*, then we can take $C_1=C_2=C_1'$ and *b* is adjacent to a reticulation. $\square$

We are now ready to prove Theorem 5.1.

### Proof of Theorem 5.1

For the first direction of the proof, assume that *A*|*B* is a CE-split in *N* and $|A|, |B|\ge 2$. Let $a_1,a_2 \in A$, $b_1,b_2 \in B$, all pairwise distinct and let $Y = \{a_1,a_2,b_1,b_2\}$. Recall that $N|_{Y}$ is obtained by applying the suppression operation to $N_{\wedge Y}$ and so the leaf set of $N|_{Y}$ is therefore *Y*. Moreover, and CE-split in *N* is also a CE-split in $N_{\wedge Y}$, and all suppression operations preserve CE-splits (but not the number of corresponding cut-edges). Hence, $\{a_1,a_2\}|\{b_1,b_2\}$ is a CE-split in $N|_{\{a_1,a_2,b_1,b_2\}}$.

To see the reverse direction, we use induction on the number of non-trivial CE-splits in *N*. The base case is that *N* is simple. To see this case, note that if *A*|*B* is a trivial split of *X* then it is certainly a CE-split in *N*. So assume that *A*|*B* is not a trivial split of *X*. We claim that if *A*|*B* is not a CE-split in *N* then there exist $a_1, a_2 \in A$, $b_1,b_2 \in B$ such that $\{a_1,a_2\}|\{b_1,b_2\}$ is not a CE-split in $N|_{\{a_1,a_2,b_1,b_2\}}$. By contraposition, this completes the proof of this direction for the base case.

To see the claim, assume that *A*|*B* is not a CE-split in *N*. By Lemma 5.5, there exist excellent cycles $C_1, C_2$ and leaves $a_1 \in A, b_1 \in B$ such that $C_1, C_2$ overlap, $a_1$ belongs to $C_1$ and $b_1$ belongs to $C_2$. In addition, either $C_1\ne C_2$ and $a_1$ and $b_1$ are both adjacent to a reticulation or $C_1= C_2$ and one of $a_1$ and $b_1$ is adjacent to a reticulation.

Let $a_2$ be an arbitrary element of $A\setminus \{a_1\}$ and let $b_2$ be an arbitrary element of $B\setminus \{b_1\}$ which must exist because *A*|*B* is not a trivial split of *X*. Put $Y=\{a_1,a_2,b_1,b_2\}$. We claim that $\{a_1,a_2\}|\{b_1,b_2\}$ is not a CE-split in $N|_Y$. To see this, note first that since $C_1$ and $C_2$ are excellent, both of them must have a unique sink adjacent to a leaf in $\{a_1,b_1\}$. Hence, these sinks and adjacent leaves are not deleted when constructing $N|_{\wedge Y}$ from *N*. Moreover, if $C_1\ne C_2$, then the cycles $C_1$ and $C_2$ are not suppressed by operation (PAS) to obtain $N|_Y$ because each of them has at least three vertices that each is incident with edges/arcs that do not form part of the cycle (one incident to $a_1$ or $b_1$ and two belonging to the other cycle). If $C_1=C_2$, this cycle is also not suppressed by operation (PAS) to obtain $N|_Y$ because the cycle contains at least three vertices that each is incident with edges/arcs that do not form part of the cycle (one incident to $a_1$, one incident to $b_1$ and one incident to or on a path to $a_2$). Finally, the blob containing $C_1$ and $C_2$ is not suppressed by operation (BLS) by the same argument. Hence, although the length of the cycles $C_1$ and $C_2$ may be shortened due to applied suppression operations, they still exist (and still overlap) in $N|_{\{a_1,a_2,b_1,b_2\}}$. Since $a_1$ belongs to $C_1$ and $b_1$ belongs to $C_2$, it follows that $\{a_1,a_2\}|\{b_1,b_2\}$ is not a CE-split in $N|_{\{a_1,a_2,b_1,b_2\}}$, as claimed.

Assume that the theorem holds for all semi-directed phylogenetic networks $N'$ on *X* and all bipartitions of *X* if $N'$ has strictly less CE-splits than *N* and that there exists a non-trivial CE-split *P*|*Q* in *N*.

We first show that $P\subseteq A$, or $P\subseteq B$, or $Q\subseteq A$ or $Q\subseteq B$ must hold. Assume for contradiction that this is not the case. Then *P* contains leaves $a_1\in A$ and $b_1\in B$ and *Q* contains leaves $a_2\in A$ and $b_2\in B$. Then $\{a_1,b_1\}|\{a_2,b_2\}$ is a CE-split in $N|_{\{a_1,a_2,b_1,b_2\}}$. Hence, $\{a_1,a_2\}|\{b_1,b_2\}$ is not a CE-split in $N|_{\{a_1,a_2,b_1,b_2\}}$, a contradiction.

Hence, we have that $P\subseteq A$, or $P\subseteq B$, or $Q\subseteq A$ or $Q\subseteq B$. Without loss of generality, assume that $P\subseteq A$. Let $\{u,v\}$ be a cut-edge such that deleting $\{u,v\}$ creates two connected components: one connected component $N_P$ containing *u* and all leaves from *P* and one connected component containing *v* and all leaves from *Q*. Construct a network $N'$ from $N_P$ by adding a new leaf $a^*$ and an edge $\{a^*,u\}$. Let $A'=(A\setminus P)\cup \{a^*\}$ and $B'=B$. Note that $N'$ has at least one non-trivial CE-split less than *N*. To be able to apply induction to $N'$, we need that, for any $a_1,a_2 \in A', b_1,b_2 \in B'$, $\{a_1,a_2\}|\{b_1,b_2\}$ is a CE-split in $N'|_{\{a_1,a_2,b_1,b_2\}}$. If $a^*\notin \{a_1,a_2\}$ then this is clear because $\{a_1,a_2\}|\{b_1,b_2\}$ is a CE-split in $N|_{\{a_1,a_2,b_1,b_2\}}$ and hence also in $N'|_{\{a_1,a_2,b_1,b_2\}}$. If $a^*\in \{a_1,a_2\}$ then assume without loss of generality that $a^*=a_1$. Let $c\in P$. Then $\{c,a_2\}|\{b_1,b_2\}$ is a CE-split in $N|_{\{c,a_2,b_1,b_2\}}$ and hence $\{a_1,a_2\}|\{b_1,b_2\}$ is a CE-split in $N'|_{\{a_1,a_2,b_1,b_2\}}$. Hence, by induction, $A'|B'$ is a CE-split in $N'$. It follows directly that *A*|*B* is a CE-split in *N*. $\square$

We conclude this section by noting that, since undirected phylogenetic trees are encoded by their splits, it follows from Theorem 5.1 that the blob tree of a semi-directed phylogenetic network is uniquely determined by the quarnets of the network. Stated more precisely, we have the following corollary.

### Corollary 5.6

Suppose that *N* and $N'$ are semi-directed phylogenetic networks on *X* with $Q(N)\simeq Q(N')$. Then $B(N)\cong B(N')$.

## Level-2 Networks

In this section, we combine the results from Sections 4 and 5 to prove that semi-directed level-2 networks with at least four leaves are encoded by their quarnets. For that, we will need the following lemma.

### Lemma 6.1

Let *N* be a semi-directed, strict level-*k* phylogenetic network on *X*, $|X|\ge 4$, for $k \ge 3$. Then there exists a quarnet $q\in Q(N)$ such that *q* is not level-2.

### Proof

**Fig. 13:**
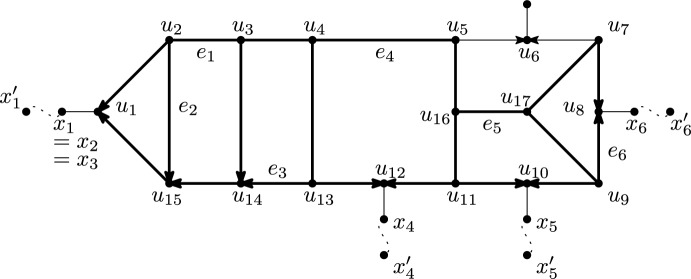
Illustration used in the proof of Lemma 6.1. The solid edges indicate the network $N_B$. The dotted edges indicate paths outside $N_B$ to leaves of *N*. Bold edges indicate the final *U*. The indicated edges $e_1,\ldots ,e_6$ are one possibility for the edges chosen in the proof of Lemma 6.1. In that case, $C_1,\ldots ,C_6$ could be the excellent cycles with vertices $(u_1,u_2,u_3,u_{14},u_{15},u_1)$, $(u_1,u_2,u_{15},u_1)$, $(u_1,u_2,u_3,u_4,u_{13},u_{14},u_{15},u_1)$, $(u_4,u_5,u_{16},u_{11},u_{12},u_{13},u_4)$, $(u_9,u_{10},u_{11},u_{16},u_{17},u_9)$ and $(u_7,u_8,u_9,u_{17},u_7)$ respectively. This leads to the quarnet $q=N|_{\{x'_1,x'_4,x'_5,x'_6\}}$, which is not level-2

Consider any blob *B* of *N* with exactly *k* reticulations. Let $N_B$ be the semi-directed, simple, strict level-*k* network obtained from *N* by deleting all vertices that are not in *B* and do not have an adjacent vertex that is in *B*.

We construct a set $A\subseteq L(N_B)$ and a set $\mathcal {C}$ of excellent cycles with $|\mathcal {C}|\ge 3$ in $N_B$ such that each $C\in \mathcal {C}$ overlaps with at least one $C'\in \mathcal {C}\setminus \{C\}$ as follows. See Figure 13 for an example.

Let $e_1$ be any edge/arc of $N_B$ between two non-leaf vertices. Then, by Lemma 5.4, there exists an excellent cycle $C_1$ in $N_B$ containing $e_1$. Let $x_1$ be the leaf of $N_B$ below the sink of $C_1$. Initialize $A=\{x_1\}$, $\mathcal {C}=\{C_1\}$ and $U=C_1$.

Repeat the following while $U\ne B$ and $|A|<4$. Let $i=|\mathcal {C}|+1$ and $e_i$ any edge/arc between two non-leaf vertices of $N_B$, such that $e_i$ is not in *U* but is incident to at least one vertex in *U*. Note that $e_i$ exists since $U\ne B$. By Lemma 5.4, there exists an excellent cycle $C_i$ in $N_B$ containing $e_i$. Note that $C_i\ne C$ for all $C\in \mathcal {C}$ and that $C_i$ overlaps with at least one $C\in \mathcal {C}$. Let $x_i$ be the leaf of $N_B$ below the sink of $C_i$. Add $C_i$ to $\mathcal {C}$, add $x_i$ to *A* (note that $x_i$ may already be in *A*, in which case *A* remains unchanged) and update *U* to be the graph union of the cycles in $\mathcal {C}$.

First suppose $|A|=4$. In this case, we have $|\mathcal {C}|\ge 4$ and hence *U* is not level-2. (To see this, note that $C_1\in \mathcal {C}$ contains a leaf reticulation, $C_2\in \mathcal {C}\setminus \{C_1\}$ either contains a different leaf reticulation or it joins $C_1$ in a different reticulation and $C_3\in \mathcal {C}\setminus \{C_1,C_2\}$ either has a leaf reticulation that is different from the leaf reticulations of $C_1$ and $C_2$ or it joins $C_1\cup C_2$ in a third reticulation.) Consider the quarnet $q_B=N_B|_A$. We now show that $q_B$ is not level-2. To see this, first recall that each $C\in \mathcal {C}$ has a unique sink with a leaf in *A* below it and sinks are not deleted by vertex suppression operations. Moreover, none of the cycles $C\in \mathcal {C}$ can be suppressed by operation (PAS). To see this, recall that *C* corresponds to an excellent cycle in $N_B$ and hence its sink is incident to a cut-edge in $N_B$. Moreover, since *C* overlaps with at least one $C'\in \mathcal {C}\setminus \{C\}$, it either has a chord (i.e., an edge/arc that is not in *C* but is incident to two vertices of *C*) or three incident edge/arcs (one where $C'$ leaves *C*, one where $C'$ joins *C* again, and one incident to the sink of *C*). In either case, *C* is not suppressed by (PAS). Finally, the blob suppression operation (BLS) is not applicable to *U* because it has at least four incident cut-edges (incident to the leaves in *A*). Hence $q_B$ is not level-2. Let $A'\subseteq X$ consist of, for each $x_i\in A$, one leaf $x'_i$ of *N* that is below $x_i$ in *N*. Then $q=N|_{A'}$ is equal to $q_B$ with each leaf $x_i$ replaced by $x_i'$. Hence, *q* is not level-2.

Now consider the case that $|A|<4$. In this case we have $U=B$ because otherwise the while loop would not have terminated. Let $A'\subseteq X$ contain, for each $x_i\in A$, one leaf $x'_i$ of *N* that is below $x_i$ in *N*. In addition, add arbitrary leaves from *X* to $A'$ until $|A'|=4$. Then $q=N|_{A'}$ contains a blob $U=B$ in which no suppression operations are applicable since *B* is a blob of *N* which is phylogenetic. Hence, *q* is not level-2 since it contains *B* which is not level-2. $\square$

We are now ready to prove the main result of this section.

### Theorem 6.2

The class of semi-directed, level-2, binary phylogenetic networks with at least four leaves is encoded by quarnets.

### Proof

Let *N* be a semi-directed level-2 phylogenetic network with at least four leaves. Let *X* be the leaf set of *N*. Let $N'$ be a semi-directed network on *X* with $Q(N)\simeq Q(N')$. We need to show that $N\cong N'$.

First we prove that $N'$ has level-2. Assume for a contradiction that $N'$ is strict level-*k* with $k\ge 3$. By Lemma 6.1, there exists a quarnet $q\in Q(N')$ that is not level-2. This leads to a contradiction since $q\in Q(N) \simeq Q(N')$ and *N* has level-2. Thus, $N'$ is a level-2 network.

We now prove that $N\cong N'$ by induction on the number *s* of nontrivial CE-splits in *N*.

If $s=0$, then *N* is a semi-directed, simple level-2 phylogenetic network on *X*. Since $Q(N)\simeq Q(N')$ it follows that $N'$ is also a semi-directed, simple, level-2 phylogenetic network. By Theorem 4.5, $N\cong N'$ follows.

So assume that $s\ge 1$. Observe that, by Theorem 5.1, $N'$ has the same CE-splits as *N*. Consider a nontrivial CE-split *A*|*B* of *N* and $N'$ (which exists since $s\ge 1$). Pick some $a\in A$ and $b\in B$ and consider the networks $N|_{A\cup \{b\}}$ and $N|_{B\cup \{a\}}$. Since $Q(N|_{A\cup \{b\}}) \simeq \{q\in Q(N) \mid L(q)\subseteq A\cup \{b\}\}$, $Q(N'|_{A\cup \{b\}}) \simeq \{q\in Q(N') \mid L(q)\subseteq A\cup \{b\}\}$, and $Q(N)\simeq Q(N')$, we have that $Q(N|_{A\cup \{b\}})\simeq Q(N'|_{A\cup \{b\}})$. If we also have $|A\cup \{b\}|\ge 4$ then it follows by induction that $N|_{A\cup \{b\}}\cong N'|_{A\cup \{b\}}$. Otherwise, we have $|A|=2$ and there exists $b'\in B$ with $b'\ne b$. It then follows directly that $N|_{A\cup \{b,b'\}}\cong N'|_{A\cup \{b,b'\}}$ (since both are quarnets) and hence that $N|_{A\cup \{b\}}\cong N'|_{A\cup \{b\}}$ (since both can be obtained from $N|_{A\cup \{b,b'\}}$ by deleting $b'$ and applying the suppression operation).

By symmetry, we also have that $N|_{B\cup \{a\}}\cong N'|_{B\cup \{a\}}$.

Since *A*|*B* is a CE-split, there exists a cut-edge $\{u,v\}$ of *N* such that the removal of it results in two connected graphs $N_A,N_B$ with leaf sets *A* and *B*, respectively. Without loss of generality, *u* is in $N_A$ and *v* is in $N_B$. Observe that, by definition, $N|_{A\cup \{b\}}$ can be obtained from $N_A$ by adding leaf *b* with an edge $\{u,b\}$. Similarly, $N|_{B\cup \{a\}}$ can be obtained from $N_B$ by adding leaf *a* with an edge $\{v,a\}$. Then, *N* can be obtained from $N|_{A\cup \{b\}}$ and $N|_{B\cup \{a\}}$ by deleting *b* and its incident edge from $N|_{A\cup \{b\}}$, deleting *a* and its incident edge from $N|_{B\cup \{a\}}$ and adding an edge $\{u,v\}$. In exactly the same way, $N'$ can be obtained from $N'|_{A\cup \{b\}}$ and $N'|_{B\cup \{a\}}$. Since $N|_{A\cup \{b\}}\cong N'|_{A\cup \{b\}}$ and $N|_{B\cup \{a\}}\cong N'|_{B\cup \{a\}}$, it follows that $N\cong N'$. $\square$

## Discussion

In this paper we have shown that the set of quarnets of a semi-directed level-2 phylogenetic network encodes the network, but that this is no longer necessarily true for level-3 networks. In addition, we proved that the blob tree of a semi-directed phylogenetic network is encoded by the quarnets of the network for any level.

There are several directions that could be of interest to be investigated next. First, it could be useful for practical applications to develop algorithms that compute semi-directed level-2 networks from collections of quarnets. As a first step in this direction it would be interesting to develop an algorithm that computes a semi-directed level-2 network from its set of quarnets (see Frohn et al. (2025) for such an algorithm for level-1). We could then adapt the algorithm to robustly deal with arbitrary collections of level-2 quarnets, similar to the Squirrel and NANUQ+ algorithms for level-1 Allman et al. (2025); Holtgrefe et al. (2025).

An $O(n^3)$-time algorithm for constructing the blob tree of a semi-directed phylogenetic network of any level from quarnets was recently developed (Frohn et al. 2025) based on the results in this paper. An interesting open problem is whether the blob tree can be reconstructed from only $O(n^2)$ quarnets and whether this is possible in $O(n^2)$ time. From a practical point-of-view it is important to develop robust blob tree construction methods. If *n* is not too big, practical algorithms could use information from all $O(n^4)$ quarnets (Allman et al. 2024; Holtgrefe et al. 2025), but when considering real data such methods currently struggle to decide how resolved to make the blob tree.

In another direction, it could be interesting to study *inference rules* for semi-directed quarnets. For phylogenetic trees, inference rules have been studied for some years, where they are used to infer new trees from collections of trees (see e.g. [Semple and Steel (2003), Section 6.7). In Huber et al. (2018), certain inference rules are given for level-1 undirected networks on four leaves, and it would be interesting to see whether similar rules can be developed for the semi-directed case. In a related direction, it could also be worth investigating approaches for deciding whether or not an arbitrary collection of quarnets (i.e. not necessarily one quarnet for each quartet of leaves) can be displayed by some semi-directed phylogenetic network. Note, however, that it is NP-complete to decide whether there is a tree that displays an arbitrary collection of quartet trees (Steel 1992).

Although we have shown that semi-directed level-3 networks are, in general, not encoded by their quarnets, it could be of interest to find a maximal subclass of level-3 (or higher) networks that is encoded by quarnets. In particular, we conjecture that the class of all semi-directed binary simple level-3 networks, except for the networks $N_1,N_2$ in Figure 2 and networks that can be obtained from $N_1$ and $N_2$ by inserting leaves on the side of *a* and *b* (in any order), is encoded by quarnets.

Finally, one major challenge that remains is to develop robust ways to construct quarnets from real data. This problem has generated considerable interest in the area of algebraic geometry, where the problem of identifying level-1 quarnets using algebraic invariants arising from models of sequence evolution has yielded some positive results on network identifiability (see e.g. Gross et al. (2021)). Some recent progress has also been made in Cummings and Hollering (2025); Martin et al. (2023) for computing level-1 quarnets for real data using algebraic invariants, but extending these approaches to level-2 quarnets appears to be a challenging problem (Ardiyansyah 2021).

## Figures and Tables

**Fig. 14:**
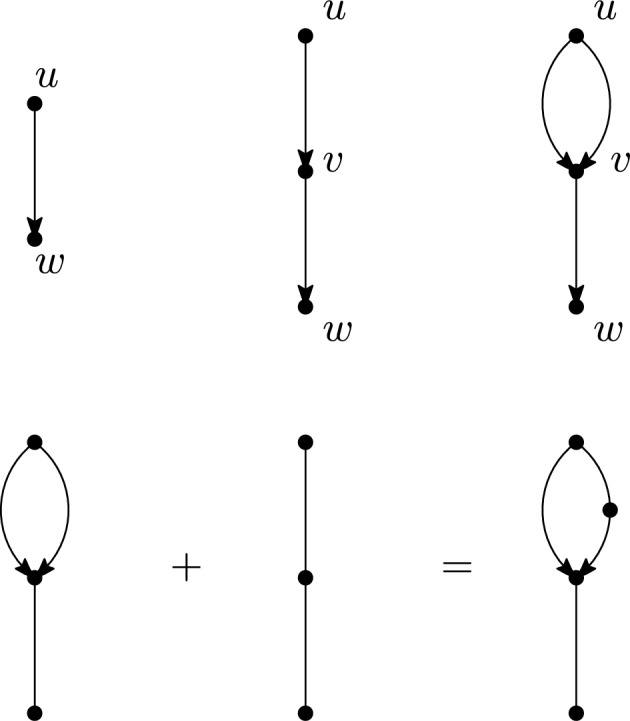
Examples of SP-graphs

**Fig. 15:**
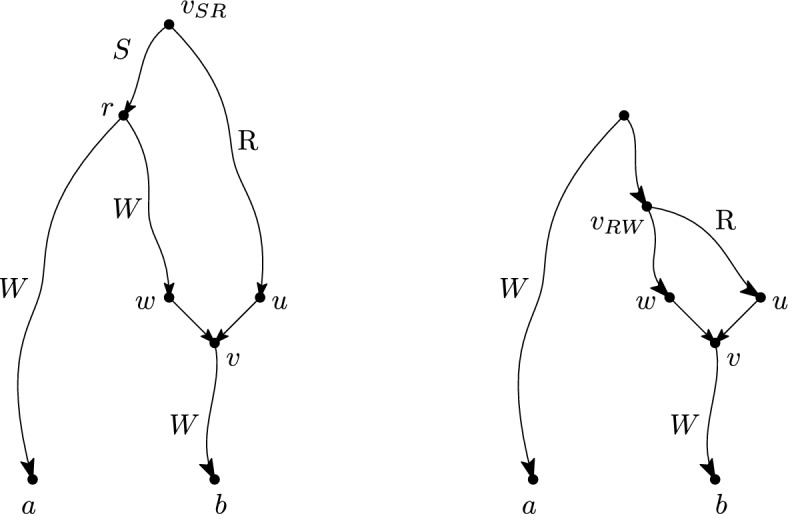
Illustrations for the proof of Lemma 3.3 for the case that *N* is directed

## Data Availability

No data was used.

## Omitted Proofs

In this appendix, we provide the previously omitted proofs for Lemmas 3.1, 3.2 and 3.3.

### Lemma

3.1. Let *N* be a semi-directed network. If $N'$ is derived from *N* by a single application of (V1), (V2), (BLS) or (PAS), then $N'$ is also a semi-directed network.

### Proof

If the operation is of type (BLS), then let *B* be an affected blob in *N*. Note that there is a corresponding blob in any rooting $N_d$ of *N*. If this blob has one incoming and one outgoing arc in $N_d$, then the same operation is also applicable to $N_d$ and applying it results in a rooting of $N'$. Otherwise, the blob corresponding to *B* has two outgoing arcs and no incoming arcs in $N_d$. Then replacing this blob with a single root vertex again gives a directed network which is a rooting of $N'$. Hence, $N'$ is semi-directed.

If the operation is of type (PAS), we claim that there exists a rooting $N_d$ of *N* such that the edge/arc that is subdivided by the root is not one of the suppressed parallel arcs (*u*, *v*). To see this, note that by definition of (PAS) vertex *u* has degree 3 and hence has an incident edge $\{u,w\}$. If there exists a rooting of *N* with the root subdividing one of the arcs (*u*, *v*), then there also exists a rooting $N_d$ of *N* with the root subdividing $\{u,w\}$. Then (PAS) is applicable to $S_d$ giving a rooting of $N'$. Hence, $N'$ is semi-directed.

If the operation is of type (V1) or (V2), we claim that, unless *N* has only three vertices, there exists a rooting $N_d$ of *N* such that the edge/arc that is subdivided by the root is not incident to the suppressed vertex *v*. Let $\{u,v\}$ be an edge incident to *v* such that *u* is not a leaf (which exists unless *N* has exactly three vertices). Then *u* has at least one other incident edge/arc, say to vertex *p*. If there exists a rooting of *N* with the root subdividing one of the edge/arcs incident to *v*, then there also exists a rooting $N_d$ of *N* with the root subdividing the edge/arc between *u* and *p*. Then suppression operation (V3) is applicable to $N_d$ giving a rooting of $N'$. Hence, $N'$ is semi-directed. Finally, if *S* contains exactly three vertices then $N'$ consists of two vertices connected by an edge and it is clear that $N'$ is again semi-directed. $\square$

We now turn our attention to the proof of Lemma 3.2, i.e. that $\textsc {Supp}(N)$ is well-defined on directed and semi-directed networks. For this, we need some additional definitions and lemmas.

Let *N* be a network. Call a subgraph *Z* of *N* a subgraph *from u to w* if all arcs/edges in *Z* are on some semi-directed path from *u* to *w*, and *N* has no arcs/edges incident to $V(Z)\setminus \{u,w\}$ except for those in *E*(*Z*). We call $Int(Z):= V(Z)\setminus \{u,w\}$ the *internal vertices of Z*. Furthermore, if *A* is a subset of the vertices of *N*, then *N*[*A*] is used to denote the subnetwork of *N* induced by *A*, i.e., the subnetwork obtained by deleting all vertices not in *A*.

We now characterize which vertices will be suppressed by the suppression operation in a directed network. We will show that these are precisely the internal vertices of subgraphs of the following type. Define the *directed SP-graphs (suppressed graphs)* as follows:

- (single arc) the graph $(V = \{u,w\}, E =\{(u,w)\})$ is a directed SP-graph from *u* to *w*.
- (parallel arcs) the graph with $V = \{u,v\}$ and parallel arcs (*u*, *v*) is a directed SP-graph from *u* to *v*.
- (series) If $Z_1 = (V_1,E_1)$ is a directed SP-graph from *u* to *v* and $Z_2 = (V_2,E_2)$ is a SP-graph from *v* to *w* with $V_1\cap V_2 = \{v\}$, then $(V_1\cup V_2, E_1 \cup E_2)$ is a SP-graph from *u* to *w*.
- (recursion) If *Z* is a directed SP-graph from *u* to *w* and $Z'$ an SP-subgraph of *Z* from $u'$ to $w'$ (i.e. $Z'$ is a subgraph of *Z* that is a directed SP-graph), where $u'$ appears before $w'$ in a directed path from *u* to *w*, then the result of replacing $Z'$ with another directed SP-graph from $u'$ to $w'$ is also a directed SP-graph from *u* to *w*.

We note without proof the following properties of a directed SP-graph *Z*: *Z* has a single vertex *u* of indegree 0 and a single vertex *w* of outdegree 0, and all arcs of *Z* are on a directed path from *u* to *w*. Every directed SP-graph with more than one arc has either a vertex of degree 2, or a pair of parallel arcs.

Define the *semi-directed SP-graphs* to be the mixed graphs that can be derived from a directed SP-graph by unorienting all arcs except for those entering reticulations. We call a mixed graph an *SP-graph* if it is a directed or semi-directed SP-graph. See Fig. 14 for examples of SP-graphs.

When *Z* is an SP-subgraph from $\rho$ to *w* in *N* for $\rho$ the root of *N*, and *Z* contains both out-arcs of $\rho$, then we say *Z* is *degenerate*.

### Lemma A.1

Let $N_1,N_2$ be networks such that $N_2$ is derived from $N_1$ by an application of (V1) or (V2) or (V3) or (PAS), and let $\{v^*\} = V(N_1)\setminus V(N_2)$.

Then for any $u,w \in V(N_2)$, it holds that $N_1$ has an SP-subgraph from *u* to *w* if and only if $N_2$ has an SP-subgraph from *u* to *w*. In particular, if *Z* is an SP-subgraph from *u* to *w* in $N_i$ for $i \in \{1,2\}$, there exists an SP-subgraph $Z'$ from *u* to *w* in $N_{3-i}$ with $V(Z)\Delta V(Z') \subseteq \{v^*\}$, and $Z'$ is degenerate if and only if *Z* is. Furthermore *w* has the same number of incoming arcs and incident edges in *Z* as in $Z'$.

### Proof

If $N_2$ is derived from $N_1$ by an application of (PAS), then for some $u^*,w^*\in V(N_1)$ (with $u^*$ not the root of *N*), there exist parallel arcs $(u^*,v^*)$ and a single arc $(v^*,w^*)$ or edge $\{v^*,w^*\}$ (because $v^*$ has degree 3). Then $N_1[\{u^*,v^*,w^*\}]$ is an SP-subgraph from $u^*$ to $w^*$ in $N_1$, and $N_2$ is derived from $N_1$ by replacing this SP-subgraph with the arc $(u^*,w^*)$ or edge $\{u^*,w^*\}$. On the other hand if $N_1$ is derived from $N_1$ by an application of (V1), (V2) or (V3), then $v^*$ has degree 2 and neighbors $u^*, w^*$, and $N_1[\{u^*,v^*,w^*\}]$ is again an SP-subgraph from $u^*$ to $w^*$ in $N_1$, and $N_2$ is derived from $N_1$ by replacing this SP-subgraph with an arc or edge from $u^*$ to $w^*$. Thus, we may now assume that $Y_1^*: = N_1[\{u^*,v^*,w^*\}]$ is an SP-subgraph from $u^*$ to $w^*$ in $N_1$, and that $N_2$ is derived from $N_1$ by replacing $Y_1$ with an arc/edge $e^*$ from $u^*$ to $w^*$. (Note that this arc/edge itself also forms an SP-subgraph from $u^*$ to $w^*$.) Note also that $e^*$ is an arc if and only if $w^*$ has in incoming arc (as opposed to an incident edge) in $Y_1^*$.

Now consider any SP-subgraph $Z_1$ from *u* and *w* in $N_1$, with $v^*\notin \{u,w\}$. If $v^*\notin V(Z_1)$ then $Z_2: = Z_1$ is also an SP-subgraph from *u* to *w* in $N_2$. Otherwise, $V(Z_1)$ contains not just $v^*$ but also $u^*$ and $w^*$ (otherwise $v^*$ is not on a path from *u* to *w*). Thus $Y_1^*$ is an SP-subgraph from $u^*$ to $w^*$ in $Z_1$. Let $Z_2$ be derived from $Z_1$ by replacing $Y_1^*$ with the arc/edge $e^*$.

Then $Z_2$ is a subgraph from *u* to *w* in $N_2$, and by construction $Z_2$ is an SP-graph with $V(Z_1)\Delta V(Z_2) \subseteq \{v^*\}$.

Conversely, consider any SP-subgraph $Z_2$ from *u* and *w* in $N_2$. If $Z_2$ does not contain $e^*$, then $Z_1:= Z_2$ is also an SP-subgraph from *u* to *w* in $N_1$. Otherwise, let $Z_1$ be derived from $Z_2$ by replacing $e^*$ with $Y_1^*$. Then $Z_1$ is a subgraph from *u* to *w* in $N_1$, and by construction $Z_1$ is an SP-subgraph with $V(Z_1)\Delta V(Z_2) \subseteq \{v^*\}$.

It remains to show that $Z_1$ is degenerate if and only if $Z_2$ is degenerate, for both constructions described above. Note that *w* has the same number of incoming arcs and incident edges in $Z_1$ as in $Z_2$. Indeed these arcs/edges are the same in both SP-subgraphs unless $w=w^*$, in which case the claim follows by comparing $Y_1^*$ with $e^*$. Note also that (for both constructions), *u* has the same degree in $Z_2$ as in $Z_1$, unless $u = u^*$ and rule (PAS) was applied, in which case *u* is not the root. It follows that $Z_2$ is degenerate if and only if $Z_1$ is degenerate. $\square$

We can now prove the following lemma, nwhich we will use to show that exhaustively applying (PAS), (V1), (V2), (V3) in any order results in the same network:

### Lemma A.2

Let $N_1 = N, N_2, \dots , N_m$ be a sequence of networks, $m \ge 2$, such that $N_{i+1}$ is derived from $N_i$ by an application of (V1) or (V2) or (V3) or (PAS), for each $i \in \{1,\dots , m-1\}$, and such that (V1),(V2),(V3) and (PAS) do not apply to $N_m$. Then

1. For each vertex *v* of *N*, $v \in V(N)\setminus V(N_m)$ if and only if *v* is an internal vertex of some non-degenerate SP-subgraph in *N*;
2. For each $u,w \in V(N_m)$, there is a single arc $(u,w) \in E(N_m)$ if and only if there is a non-degenerate SP-subgraph from *u* to *w* in *N* which ends in an arc.
3. For each $u,w \in V(N_m)$, there is a single edge $(u,w) \in E(N_m)$ if and only if there is a non-degenerate SP-subgraph from *u* to *w* in *N* which ends in an edge.
4. For each $u,w \in V(N_m)$, there are parallel arcs $(u,w) \in E(N_m)$ if and only if there is a minimal degenerate SP-subgraph in *N* that is a degenerate SP-subgraph from *u* to *w*.

### Proof

Note that, for any vertex *v* removed by an application of (PAS) or (V3) on some nnetwork $N'$, *v* is part of an SP-subgraph *Z* from *u* to *w* in $N'$, where *u* and *w* are the parent and child of *v* respectively. Furthermore *Z* is non-degenerate (as we do not apply (PAS) when *u* is the root).

To prove Statement 1, first suppose that $v \in V(N)\setminus V(N_m)$, and let *i* be the unique integer for which $v \in V(N_i)\setminus V(N_{i+1})$. Then *v* was removed by an application of (PAS) or (V3) on $N_i$, and so *v* is part of a non-degenerate SP-subgraph $Z_i$ from *u* to *w* in $N_i$. If $i > 1$, then by Lemma A.1 there exists a non-degenerate SP-subgraph $Z_{i-1}$ from *u* to *w* in $N_{i-1}$, with $V(Z_{i-1}) \subseteq V(Z_i)$. Thus *v* is also an internal vertex of $Z_i$. Repeating this argument, we see that *v* is an internal vertex of a non-degenerate SP-subgraph from *u* to *w* in $N_1 = N$, as required.

Conversely, suppose that *v* is an internal vertex of a non-degenerate SP-subgraph from *u* to *w* in $N_1$. Note that $N_m$ has no non-degenerate SP-graphs except for those subgraphs consisting of a single arc, as otherwise one of (PAS) or (V3) would apply to $N_m$. So there exists some largest $i \in \{1,\dots , m-1\}$ such that *v* is an internal vertex of a non-degenerate SP-subgraph in $N_i$, but not in $N_{i+1}$. Let $Z_i$ be such a non-degenerate subgraph, and suppose $Z_i$ is from *u* to *w*. Let $v^*$ be the unique vertex in $V(N_i)\setminus V(N_{i+})$. Note that if $u = v^*$ then *v* is also part of a non-degenerate SP-graph from $u^*$ to *w* for $u^*$ the parent of *u*, and if $w = v^*$ then *v* is part of a non-degenerate SP-subgraph from *u* to $w^*$ for $w^*$ the child of *w*.

Thus, we may assume neither *u* nor *w* is $v^*$. So Lemma A.1 implies that $N_{i+1}$ also has a non-degenerate SP-graph from *u* to *w*, with $V(Z_{i+1}) \supseteq V(Z_i)\setminus \{v^*\}$. As *v* cannot be in $Z_{i+1}$ by choice of *i*, it follows that $v = v^*$, and so $v \notin V(N_m)$, as required.

We now have that $v \in V(N_m)$ if and only if *v* is not part of a non-degenerate SP-graph in *N* (Statement 1). It remains to consider the arcs and edges of $N_m$ (Statements 2-4).

By Lemma A.1, for any $u,w \in V(N_{i+1})$, there is a non-degenerate SP-subgraph *Z* from *u* to *w* in $N_{i+1}$ if and only if there is a non-degenerate SP-subgraph from *u* to *w* in $N_i$, for all $i \in \{1,\dots , m-1\}$. It follows that there is a non-degenerate SP-subgraph from *u* to *w* in $N_m$ if and only if there is a non-degenerate SP-subgraph from *u* to *w* in $N_1 = N$. But the only non-degenerate SP-subgraphs in $N_m$ are arcs and edges. Moreover, the SP-subgraph from *u* to *w* ends in an incoming arc of *w* if and only if *Z* ends in an incoming arc of *w*. So $N_m$ has an arc from *u* to *w* if *Z* ends in an arc, and $N_m$ has an edge between *u* and *w* if *Z* ends in an edge. nThis concludes the proof of Statements 2 and 3.

Finally, again by Lemma A.1, there is a degenerate SP-subgraph from *u* to *w* in $N_m$ if and only if there is a degenerate SP-subgraph from *u* to *w* in $N_1 = N$. Statement 4 now follows since, in $N_m$ the only degenerate nSP-subgraphs are pairs of parallel arcs. $\square$

### Lemma

3.2. $\textsc {Supp}(N)$ is well-defined nfor any network *N*.

### Proof

Let $N_1$ be the network derived from *N* by applying a n(BLS) operation to every

blob with at most two incident edge/arcs in *N*. Note that suppressing one blob does not affect the other blobs in the network, and so $N_1$ nis well-defined.

Considering the definition of $\textsc {Supp}(N)$, it remains to show that starting with $N_1$ and exhaustively applying the operations (PAS), (V1), (V2), (V3) will always result in the same network.

To see this, let $$N_1, N, N_2, \dots , N_m$$ and $$N'_1 = N_1, N, N'_2, \dots , N'_{m'}$$ be two sequences of networks, such that $$N_{i+1}$$ (respectively, $$N'_{i+1}$$) is derived from $$N_i$$ ($$N'_i)$$ by an application of (V1) or (V2) or (V3) or (PAS), for each $$i \in \{1,\dots , m-1\}$$ ($$i \in \{1,\dots , m'-1\}$$), and such that (V1),(V2),(V3) and (PAS) do not apply to $$N_m$$ ($$N'_{m'}$$). By applying Lemma A.2 to $$N_m$$ and $$N'_{m'}$$, we see that $$N_m$$ and $$N'_{m'}$$ have exactly the same vertices (Statement 1 of Lemma A.2), arcs (Statement 2 of Lemma A.2), edges (Statement 3 of Lemma A.2) and parallel arcs (Statement 4 of Lemma A.2). Thus, $$N_m$$ and $$N'_{m'}$$ are the same network, and so $$\textsc {Supp}(N)$$ is well-defined. $$\square $$

### Lemma

3.3. Consider a network *N* on *X*, leaves $a,b\in X$ and a reticulation *v* with parents *u*, *w*. If *v* is on a $\wedge$-path in *N* between *a* and *b*, then *u* is on a $\wedge$-path in *N* between *a* and *b*.

### Proof

First suppose that *N* is a directed network.

Consider any $\wedge$-path *W* between *a* and *b* containing *v*. It contains at least one of *u* and *w*. If *W* contains *u* then the lemma holds. Hence, suppose that *W* does not contain *u* and hence traverses the arc (*w*, *v*). Assume without loss of generality that arc (*w*, *v*) is traversed on the part of *W* directed towards *b*. Consider any directed path *R* from the root of *N* to *u* (which exists as *N* is assumed to be directed).

First suppose that *R* is disjoint from *W*. Let *r* be the vertex of *W* such that *W* consists of directed paths from *r* to *a* and *b*. Then consider a directed path *S* from the root to *r*. Let $v_{SR}$ be the last common vertex of *S* and *R*. Then a $\wedge$-path between *a* and *b* containing *u* can be obtained by following *W* from *a* to *r*, then following *S* to $v_{SR}$, following *R* to *u*, following the arc (*u*, *v*), and finally following *W* from *v* to *b*. See Figure 15 (left) for an example.

Now suppose *R* intersects *W*. Let $v_{RW}$ be the last vertex of *R* that is on *W*. Then a $\wedge$-path between *a* and *b* containing *u* can be obtained by following *W* from *a* to $v_{RW}$, then following *R* to *u*, following the arc (*u*, *v*), and finally following *W* from *v* to *b*. See Figure 15 (right) for an example.

It remains to consider the case that *N* is semi-directed. Consider any rooting *D* of *N*. If *D* contains arc (*u*, *v*), then *u* is on a $\wedge$-path in *D* between *a* and *b* by the first part of the proof (for directed networks). Hence, *u* is on a $\wedge$-path in *N* between *a* and *b*.

Otherwise, *D* contains arcs $(\rho ,u),(\rho ,v)$. Then, $\rho$ is on a $\wedge$-path in *D* between *a* and *b* by the first part of the proof. Hence, *u* is on a $\wedge$-path in *N* between *a* and *b*. $\square$

## Footnotes

1 Such paths were called “up-down paths” in Jingcheng and Ané (2023), but we use $\wedge$-paths to avoid confusion with an earlier notion of up-down paths that contain only arcs (Bordewich and Semple 2016; Murakami et al. 2019).
